# A mixed filter algorithm for sympathetic arousal tracking from skin conductance and heart rate measurements in Pavlovian fear conditioning

**DOI:** 10.1371/journal.pone.0231659

**Published:** 2020-04-23

**Authors:** Dilranjan S. Wickramasuriya, Rose T. Faghih

**Affiliations:** Department of Electrical and Computer Engineering, University of Houston, Houston, Texas, United States of America; Universita degli Studi di Pisa, ITALY

## Abstract

Pathological fear and anxiety disorders can have debilitating impacts on individual patients and society. The neural circuitry underlying fear learning and extinction has been known to play a crucial role in the development and maintenance of anxiety disorders. Pavlovian conditioning, where a subject learns an association between a biologically-relevant stimulus and a neutral cue, has been instrumental in guiding the development of therapies for treating anxiety disorders. To date, a number of physiological signal responses such as skin conductance, heart rate, electroencephalography and cerebral blood flow have been analyzed in Pavlovian fear conditioning experiments. However, physiological markers are often examined separately to gain insight into the neural processes underlying fear acquisition. We propose a method to track a single brain-related sympathetic arousal state from physiological signal features during fear conditioning. We develop a state-space formulation that probabilistically relates features from skin conductance and heart rate to the unobserved sympathetic arousal state. We use an expectation-maximization framework for state estimation and model parameter recovery. State estimation is performed via Bayesian filtering. We evaluate our model on simulated and experimental data acquired in a trace fear conditioning experiment. Results on simulated data show the ability of our proposed method to estimate an unobserved arousal state and recover model parameters. Results on experimental data are consistent with skin conductance measurements and provide good fits to heartbeats modeled as a binary point process. The ability to track arousal from skin conductance and heart rate within a state-space model is an important precursor to the development of wearable monitors that could aid in patient care. Anxiety and trauma-related disorders are often accompanied by a heightened sympathetic tone and the methods described herein could find clinical applications in remote monitoring for therapeutic purposes.

## Introduction

Human emotions represent complex processes within the nervous system. Changes in emotion manifest themselves through a number of physiological means. For instance, the human fear response, which can be activated when the brain interprets external environmental stimuli as posing a threat to survival, can cause an elevation in blood pressure, heart rate and sweating, preparing the body for action [1]. Emotions play an important role in our everyday lives as well, and are essential to self-expression, social interaction and decision-making. Much work throughout the years has aided our understanding of the neural correlates of emotion and the hemodynamic and electrical responses that accompany changes thereof [2–5]. High-level neural processes such as cognition, emotion, motivation etc. are, however, not directly observed. Nevertheless, the physiological and biochemical changes that accompany these neural processes are observable, and provide a means for their estimation. This window to neural state estimation is one that can have important implications for wearable monitoring. To illustrate, anxiety disorders often include symptoms of excessive fear and worry [6], and there is a heightened level of sympathetic nervous system activation in these patients [7]. The elevated sympathetic activation in trauma-related disorders such as post-traumatic stress disorder (PTSD) has also been noted in the literature [8, 9]. Increased sympathetic drive gives rise to measurable biosignal changes, particularly in response to certain stimuli/cues (e.g. elevated heart rate and facial electromyography [EMG] responses to trauma-related cues in PTSD patients [10]). An index of sympathetic arousal extracted conveniently from physiological signals could aid in the management of neuropsychiatric disorders involving pathological fear and anxiety.

Anxiety disorders are among the most prevalent form of mental disorder [11]. They involve high costs both to the individual patient and to society in general [12, 13]. Anxiety disorders are often accompanied by behavioral symptoms such as difficulty concentrating, situational avoidance, irritability and restlessness [6]. While fear and anxiety are part of the normal human experience, they nonetheless have the potential to grow disproportionately to the perceived threat and persist over time; this necessitates medical intervention. The neural circuitry underlying fear acquisition and the mechanisms involved thereof have long been considered crucial to the understanding of anxiety disorders and the development of treatment options [14, 15]. Of particular interest in fear learning and extinction has been the study of Pavlovian conditioning. Here, a subject learns an association between a biologically-relevant stimulus and a neutral cue through repeated pairing [16]. The paradigm originated with experiments conducted by the Russian scientist Ivan Pavlov in the early part of the 20^th^ century [16]. In his classic experiment, Pavlov repeatedly paired the ringing of a bell with food, eventually causing a dog to salivate merely at the ringing of the bell [17]. In this particular case, the food was named as the unconditioned stimulus (US) and the ringing of the bell as the conditioned stimulus (CS). Learning the association between the CS and US lies at the heart of Pavlovian conditioning; eventually the CS alone will begin to elicit the biological response typically associated with the US. In fear conditioning experiments, the US is unpleasant. It can take the form of a mild electric shock, a loud sound, an aversive image or a blast of air to the throat [17, 18]. Pavlovian fear conditioning has been examined in both human subjects and animal models. Additional forms of fear conditioning experiments arose later. These include differential conditioning and the use of more complex stimuli. In differential conditioning, there are two types of conditioned stimuli—CS+ and CS-. The CS- is never associated with the US. The CS+ may be associated fully or partially with the US. The CS+ can also be chosen to reinforce the threat of the US (e.g. the image of a fearful face may be used as the CS+ and a neutral face as the CS-). Anxiety disorders and PTSD are thought to involve a pathological dysregulation of the individual fear response and its related neural circuitry—particularly with regard to fear extinction [19]. Pavlovian fear conditioning experiments conducted on these patient populations have also helped shed light on the specific brain regions that may be involved in this dysregulation (e.g. [20–22]).

Skin conductance (or equivalently skin resistance or potential) has been the most commonly measured physiological signal in human fear conditioning experiments [18]. Changes in the conductivity of the skin occur due to salty sweat secretions and can be easily measured with electrodes placed on the fingers or palms of a subject. Skin conductance increases with sympathetic nervous system activation and is responsive to emotional arousal [17]. A skin conductance signal comprises of both a slow-varying tonic component and a faster varying phasic component [23]. The phasic component comprises of a series of individual skin conductance responses (SCRs). Each SCR occurs due to the expulsion of sweat in response to a burst of sudomotor neural activity [24, 25] which in turn may occur due to arousing stimuli [26]. In a typical fear conditioning experiment, the CS+ cues increasingly begin to elicit SCRs (which are typically elicited by the US) causing a rise in the skin conductance signal [27]. Heart rate is yet another measure of autonomic activity commonly used in Pavlovian fear conditioning [17], and several studies have examined the heart rate response in these experiments (e.g. [28–31]). Differences in these autonomic responses have also been noted between healthy subjects and neuropsychiatric patients (e.g. PTSD [32]). The fear-potentiated startle reflex is yet another measurement commonly acquired in fear conditioning experiments [33–35]. This is usually measured with the aid of EMG sensors placed around the eye to capture blinks. The startle reflex is more responsive to negative valence US than it is to neutral or positive valence stimuli [18, 36] (valence refers to the pleasure-displeasure or positive-negative axis of emotion [37]). Direct measures of the central nervous system have also been analyzed in fear conditioning experiments. Examples include cerebral blood flow from functional magnetic resonance imaging [27] and electroencephalography (EEG) [38].

While many different signals have been examined during fear conditioning, their responses have largely been analyzed separately. Here we seek to extract a single underlying sympathetic arousal state that gives rise to the observed variations in autonomic responses. We use a state-space formulation to do so. The arousal state that gives rise to the measurable changes in skin conductance and heart rate is unobserved. Now sympathetic nerve fibers innervate the sweat glands [39], and we select three commonly used skin conductance features of arousal that are generated by the tiny sweat secretions [40]. We also use heart rate which is related to arousal [41]. We relate these physiological markers to the unobserved sympathetic arousal state probabilistically and derive a Bayesian filter for state estimation. The filter is applied within an expectation-maximization (EM) framework that simultaneously estimates arousal and recovers unknown model parameters.

In a recent work [42], we developed a state-space model and corresponding Bayesian filter for estimating sympathetic arousal from the three skin conductance features just referred to. Here we extend the model to include a spiking-type observation from electrocardiography (ECG) signals. The current method therefore combines information from both the skin and the heart for estimating arousal. Our work can also be seen as a contribution from a state-space modeling viewpoint since it is an extension to [43]. Coleman et al. [43] developed a state-space model to estimate a cognitive learning state based on observing a binary correct/incorrect response variable, a continuous-valued reaction time variable and a neural spiking signal (characterized by a conditional intensity function [CIF]) in each trial of a multi-trial behavioral experiment involving a non-human primate. Our current state-space model incorporates one binary variable, two continuous-valued variables and a different form of the CIF—one which is more suited to heart rate. The following section describes our methodology. We thereafter provide results on both simulated and experimental data. We finally conclude with a discussion of our results and note future directions of research. State-space methodologies to track internal brain dynamics could ultimately lead to the development of convenient wearable monitors for long-term patient care. PTSD, for instance, is often accompanied by symptoms of hyperarousal [44], and a wearable device for estimating arousal may be helpful in tracking the status of a patient over time [45].

## Materials and methods

### Data

**Fig 1:**
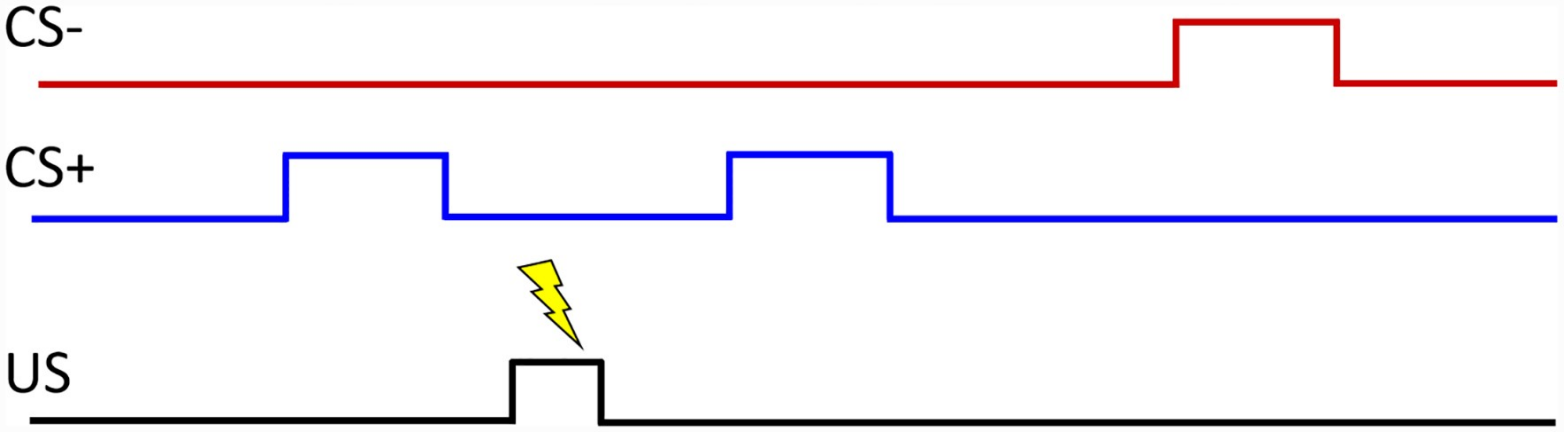
General layout of a trace fear conditioning experiment. A typical fear conditioning experiment consists of two types of cues (CS+ and CS-) and a US. The CS- is never accompanied by the US. In the data set used here, the CS+ was accompanied by the US (electric shock) only 50% of the time, and blue and red rectangles were used as the CS+ and CS- cues. Other experimental paradigms are also possible (for instance, where the CS+ is always accompanied by the US or where the CS+ reinforces the unpleasant US). In trace conditioning, there is a gap between the time when the CS+ stimulus ends and when the US begins. The figure was adapted from [17].

We used the “PsPM-TC: SCR, ECG, EMG and respiration measurements in a discriminant trace fear conditioning task with visual CS and electrical US” data set [46]. The data set is described in detail in [28, 47, 48], and is publicly available through the Zenodo repository. The original experiment involved 23 subjects (13 males, 10 females, age 23.8±3.0 years) from whom four subjects were discarded [28]. The data set that is available online contains physiological signal measurements from the other 19 healthy subjects who participated in the trace fear conditioning task. In trace fear conditioning, as opposed to delay fear conditioning, there is a time gap between the termination of the CS+ cue and the US onset [17]. Blue and red rectangles on a computer screen were used as the CS+ and CS- visual cues. The US was a series of 0.2 ms square electrical pulses applied at a frequency of 10 Hz for a duration of 0.5 s to the subject’s forearm using a pin-cathode/ring-anode electrode configuration. The stimulation intensity was set to approximately 90% of each subject’s pain threshold following a two-step procedure. Skin conductance was recorded from Ag/AgCl cup electrodes placed on the thenar/hypothenar of each subject’s non-dominant hand and ECG was likewise recorded using Ag/AgCl electrodes placed on the limbs. Only 50% of the CS+ trials were accompanied by the US. The general layout of a trace fear conditioning experiment is shown in Fig 1. Skin conductance and ECG can be contaminated by various sources of noise including motion artifacts and powerline noise. We analyzed data from 12 subjects for whom information could be extracted from the signals where low to moderate noise contamination was present. We relabeled the original subject numbers with participant numbers.

### Preprocessing

Skin conductance is a low-bandwidth signal and we first lowpass filtered the data at 0.5 Hz and then downsampled to 4 Hz. Cut-off frequencies as low as 0.4 Hz have also been used in the literature when filtering skin conductance data [49]. We next decomposed each skin conductance signal *z*_*k*_ into its constituent tonic (*s*_*k*_) and phasic components (${\tilde{r}}_{k}$) using cvxEDA [50]. We also detected ECG R-peaks using MATLAB’s *findpeaks* function and manually corrected erroneous heartbeats.

### State-space model

Random walks and first-order autoregressive (AR) models have frequently been used to capture the evolution of unobservable neural states across time (e.g. learning [43, 51–53], sleep [54] and neural states underlying spiking activity [55]). We assume that sympathetic arousal *x*_*k*_ evolves with time following the model in [55].

$$\begin{array}{cc} x_{k} & =\rho x_{k-1}+\alpha I_{k}+\varepsilon_{k}, \end{array}$$ (1)

where $\varepsilon_{k}∼N\left(0,\sigma_{\varepsilon }^{2}\right)$ and *I*_*k*_ is an indicator function representing external stimuli. *ρ* and *α* are coefficients to be determined. We take four different observations to estimate the unobserved sympathetic arousal state—three from skin conductance and one from ECG.

### Skin conductance

As noted earlier, sympathetic nerve fibers in the autonomic nervous system innervate the sweat glands [39]. Consequently, skin conductance, which varies based on sweat secretions, functions as an indicator of sympathetic arousal [56]. Several different skin conductance features have been notably used in the literature as indices of arousal. Firstly, the rate at which SCRs appear has been taken as an index of arousal—the higher the arousal, the higher the rate of SCR occurrence. SCR rate has been used as a marker of arousal in experiments involving thought suppression [57], alcohol craving [58] and audio processing [59]. Secondly, SCR amplitude has been considered an index of arousal as well. This has been used in studies involving emotional visual stimuli [60] and sounds [61]. Finally the tonic level has been used as an index of arousal in several studies. Examples include biofeedback tasks [62], antisocial behavior [63] and the presentation of visual stimuli [64]. SCR rate, SCR amplitude and the tonic level have been the three most commonly reported skin conductance markers of autonomic activity in the literature [40].

We first consider the appearance of SCRs. SCRs can be detected as phasic peaks that exceed a threshold between 0.01–0.05 μS [23]. We assign *m*_*k*_ = 1 or *m*_*k*_ = 0 based on whether or not an SCR occurred at the *k*^th^ time index using a threshold of 0.015 μS. The 0.015 μS threshold is selected similar to our previous work in [42] to provide a balance between detecting SCR peaks and avoiding the detection of noise as SCRs. The occurrence of SCRs follows a Bernoulli distribution with a density function $p_{k}^{m_{k}}{\left(1-p_{k}\right)}^{1-m_{k}}$ where *p*_*k*_ is the probability that *m*_*k*_ = 1. Therefore, we relate sympathetic arousal to the occurrence of SCRs using the theory of generalized linear models. We use a logit transformation following the suggestion in [65].

$$\begin{array}{cc} \log(\frac{p_{k}}{1-p_{k}})=\beta_{0}+\beta_{1}x_{k} & \Rightarrow p_{k}=\frac{1}{1+e^{-(\beta_{0}+\beta_{1}x_{k})}}, \end{array}$$ (2)

where *β*_0_ and *β*_1_ are regression coefficients to be determined. Therefore,

$$\begin{array}{cc} P(m_{k}|x_{k}) & =p_{k}^{m_{k}}{\left(1-p_{k}\right)}^{1-m_{k}}={\left[\frac{1}{1+e^{-(\beta_{0}+\beta_{1}x_{k})}}\right]}^{m_{k}}{\left[\frac{e^{-(\beta_{0}+\beta_{1}x_{k})}}{1+e^{-(\beta_{0}+\beta_{1}x_{k})}}\right]}^{1-m_{k}}. \end{array}$$ (3)

Secondly, we consider the continuous-valued tonic skin conductance level *s*_*k*_ which is also known to be related to arousal [66]. Other neural state estimation methods (e.g. [43, 52]) have assumed linear relationships between continuous-valued observations and the latent state to be determined. We too take *s*_*k*_ to be linearly related to *x*_*k*_.

$$\begin{array}{cc} s_{k} & =\delta_{0}+\delta_{1}x_{k}+w_{k}, \end{array}$$ (4)

where $w_{k}∼N\left(0,\sigma_{w}^{2}\right)$ captures sensor noise and modeling error, and *δ*_0_ and *δ*_1_ are regression coefficients to be determined.

**Fig 2:**
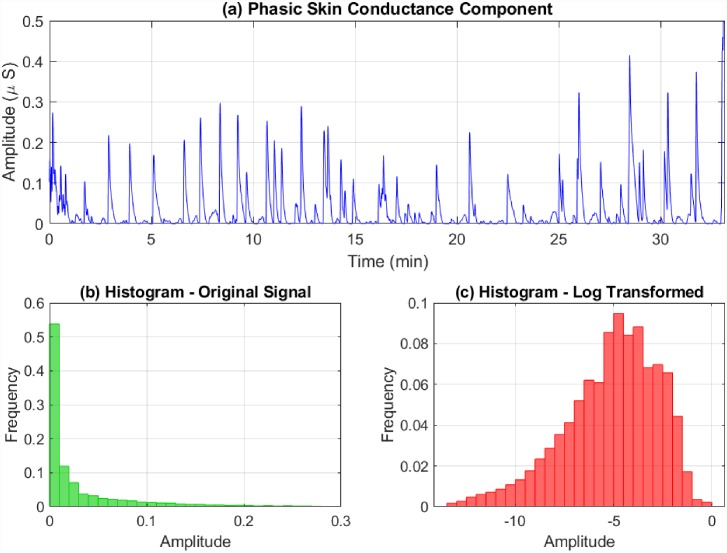
Phasic skin conductance skew correction. The phasic component of a skin conductance signal fluctuates more rapidly than its tonic counterpart and has a positively skewed amplitude histogram. The upper sub-panel depicts the phasic skin conductance from participant 1 extracted using cvxEDA. The lower sub-panels show the the amplitude histograms before and after a log transformation.

**Fig 3:**
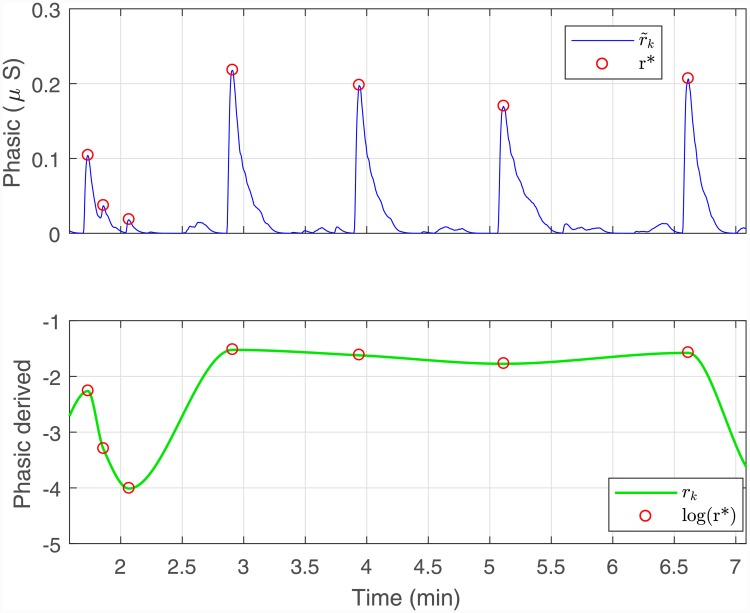
Extraction of the phasic-derived component *r*_*k*_ from ${\tilde{r}}_{k}$. The amplitudes of each of the SCRs are related to sympathetic arousal. Therefore, we derive an artificial phasic-related component by detecting peaks and then interpolating over the log values of the peak amplitudes. The figure shows a zoomed-in section illustrating the derivation.

**Fig 4:**
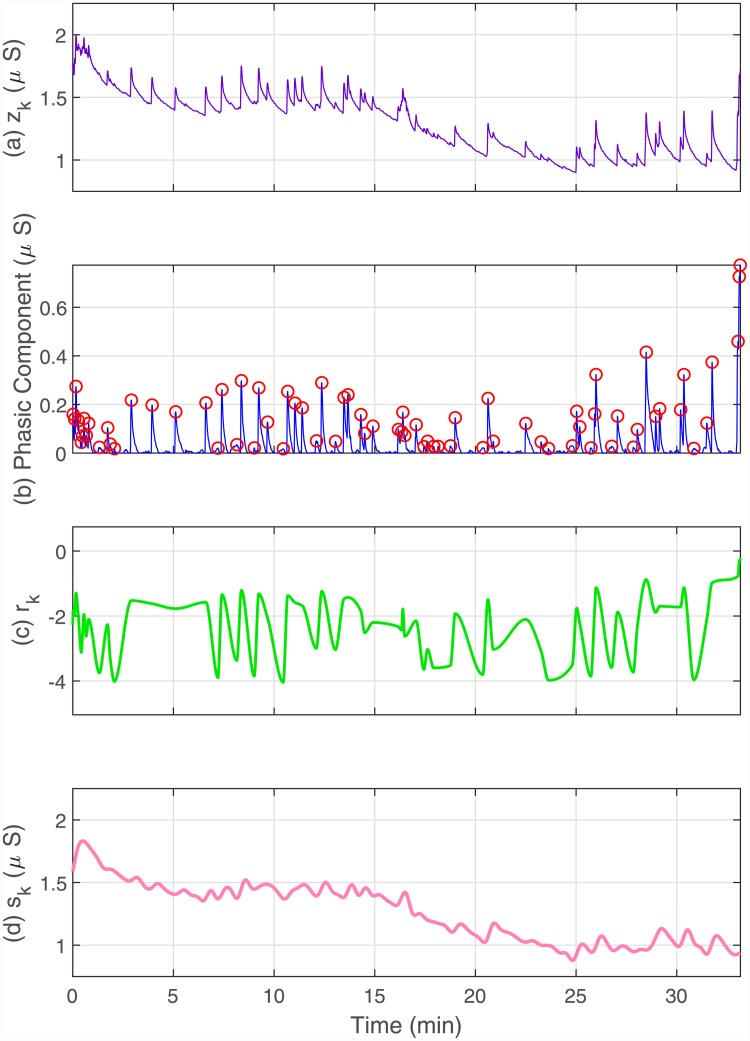
Constituent components of a skin conductance signal. The sub-panels from top to bottom respectively depict, (a) the skin conductance signal *z*_*k*_; (b) the phasic component with the detected SCR peaks; (c) the phasic-derived component *r*_*k*_; (d) the tonic component *s*_*k*_. *m*_*k*_ = 1 or *m*_*k*_ = 0 is assigned based on whether or not an SCR occurred at the *k*^th^ time-point. We make use of the observations *m*_*k*_, *r*_*k*_ and *s*_*k*_ at each time point to estimate *x*_*k*_.

We next consider the rapidly-fluctuating phasic component ${\tilde{r}}_{k}$. Two main aspects are to be noted regarding the phasic component. Firstly, its distribution is skewed. A logarithmic or square-root transformation is commonly suggested to correct skew in skin conductance measures [67]. Here we apply a logarithmic transformation (Fig 2). Secondly, it is the amplitudes of the SCRs that are considered to be related to sympathetic arousal [68]. Therefore, we derive an artificial signal *r*_*k*_ by interpolating over the SCR peaks and the first and last values of the log transformed ${\tilde{r}}_{k}$. These two steps can also be combined and expressed mathematically as follows. Taking

$$\begin{array}{cc} r^{*} & =\left\{{\tilde{r}}_{1},{\tilde{r}}_{K}\right\}\cup \left\{{\tilde{r}}_{k}|m_{k}=1\right\} \end{array}$$ (5)

to denote the phasic SCR peaks along with the first and last values, *r*_*k*_ is derived by applying a cubic interpolation over log r* (Fig 3). The positive skew of the SCR amplitudes is one that has been noted in the literature and the logarithmic transformation is often used for correction [69]. Similar to the case of *s*_*k*_, we assume a linear relationship between *r*_*k*_ and *x*_*k*_ as well.

$$\begin{array}{cc} r_{k} & =\gamma_{0}+\gamma_{1}x_{k}+v_{k}, \end{array}$$ (6)

where the coefficients *γ*_0_ and *γ*_1_ are similar to *δ*_0_ and *δ*_1_, and $v_{k}∼N\left(0,\sigma_{v}^{2}\right)$ represents a noise term similar to *w*_*k*_. $M^{K}=\left\{m_{1},m_{2},\ldots ,m_{K}\right\}$, $R^{K}=\left\{r_{1},r_{2},\ldots ,r_{K}\right\}$ and $S^{K}=\left\{s_{1},s_{2},\ldots ,s_{K}\right\}$ form the complete series of skin conductance observations (Fig 4).

### Heart rate

We next wish to extract an ECG biomarker for estimating sympathetic arousal. Now both the sympathetic and parasympathetic branches of the autonomic nervous system regulate heart rate. The sympathetic nervous system increases heart rate and the force of contraction via the neurotransmitter norepinephrine [70]. In contrast, parasympathetic activation causes the release of actylcholine at the heart and has the opposite effect. Beat-to-beat variations in RR-intervals, known as heart rate variability (HRV), reflect these changes in sympathetic and parasympathetic control on the heart. In this model, we relate sympathetic arousal to heart rate. Studies in animal models have shown that the stimulation of autonomic nerve fibers leading to the heart results in an almost linear relationship between stimulation frequency and RR-intervals [71, 72]. Based on findings in these studies, we select a linear model to capture the relationship between RR-intervals and sympathetic arousal *x*_*k*_.

Heartbeats occur due to the depolarization of cells in the heart’s sinoatrial (SA) node which subsequently propagates throughout the atria and ventricles. The rise in the membrane potentials in the SA node cells can be modeled as a Gaussian random walk with drift [73, 74]. Consequently, the times between successive ventricular contractions can be modeled using the inverse Gaussian probability density model. Barbieri et al. [73, 75] successfully used a history-dependent inverse Gaussian (HDIG) probability density function to model RR-intervals. If *L* consecutive R-peaks occur at times *u*_*l*_ within (0, *T*] such that 0 < *u*_1_ < *u*_2_ < … < *u*_*L*_ ≤ *T*, and *h*_*l*_ = *u*_*l*_ − *u*_*l*−1_ is the *l*^th^ RR-interval, then the HDIG density function for RR-intervals at *t* > *u*_*l*_ is,

$$\begin{array}{cc} g(t|u_{l}) & =\sqrt{\frac{\theta_{q+1}}{2\pi {\left(t-u_{l}\right)}^{3}}}\;\text{exp}\{\frac{-\theta_{q+1}{{\left[t-u_{l}-\hat{\mu }\right]}^{2}}_{2}}{2\hat{\mu }}\left(t-u_{l}\right)\}, \end{array}$$ (7)

where *q* is the model order, *θ*_*q*+1_ is related to the variance and

$$\begin{array}{cc} \hat{\mu } & =\theta_{0}+\sum_{i=1}^{q}\theta_{i}h_{l-i+1} \end{array}$$ (8)

is the HDIG mean [75]. The *θ*_*i*_’s are coefficients to be determined. This model expresses the dependence of an RR-interval on its immediate history (this dependence has also led to the successful application of AR models to the analysis of HRV [76, 77]). Barbieri et al. [73] divided the time axis into bins of size Δ = 5 ms and performed local likelihood estimation to determine the *θ*_*i*_’s every Δ ms (i.e, the *θ*_*i*_’s were time-varying). These time-varying parameters capture part of the non-stationary nature of HRV that occurs due to underlying pathological and physiological reasons [78].

Based on our earlier assumption of linearity between sympathetic arousal *x*_*k*_ and the RR-intervals, we re-define a new HDIG RR-interval mean *μ* as one which depends linearly on both the immediate history and the arousal *x*_*k*_, i.e.,

$$\begin{array}{c} \mu =\theta_{0}+\sum_{i=1}^{q}\theta_{i}h_{l-i+1}+\eta x_{k}, \end{array}$$ (9)

where *η* is a coefficient to be determined. Moreover, we also assume that the *θ*_*i*_’s are fixed and that variations in sympathetic arousal account for part of the RR-interval stochasticity. According to this formulation, changes in arousal would cause the HDIG probability density function to shift to the right or to the left.

Recall that we analyze skin conductance data at a sampling frequency of 4 Hz due to its low bandwidth (sampling time *t*_*s*_ = 250 ms). Now the bin size Δ = 5 ms proposed by Barbieri et al. [73] used for the HDIG model is much smaller. There are *J* = *t*_*s*_/Δ = 50 heart rate observation bins corresponding to the *k*^th^ skin conductance sample. We index these smaller heart rate bins over *j* and generate a binary point process by assigning *n*_*k*,*j*_ = 1 or *n*_*k*,*j*_ = 0 depending on whether or not an R-peak occurred at the time. The joint density over these *J* observations is then [79]

$$\begin{array}{cc} P(n_{k,1},n_{k,2},\ldots ,n_{k,J}|x_{k}) & =e^{\sum_{j=1}^{J}\log\left(\lambda_{k,j}\Delta \right)n_{k,j}-\lambda_{k,j}\Delta }, \end{array}$$ (10)

where the CIF λ_*k*,*j*_ is

$$\begin{array}{cc} \lambda_{k,j} & ≜\frac{g(t_{k,j}|u_{k,j})}{1-\int_{u_{k,j}}^{t_{k,j}}g\left(z|u_{k,j}\right)dz}, \end{array}$$ (11)

where *u*_*k*,*j*_ is the time of occurrence of the last R-peak prior to time *t*_*k*,*j*_. $N^{K}=\left\{n_{1,1},n_{1,2},\ldots ,n_{1,J},n_{2,1},n_{2,2},\ldots ,n_{2,J},\ldots ,n_{K,1},n_{K,2},\ldots ,n_{K,J}\right\}$ form the set of heart rate observations.

### State estimation and parameter recovery

Given the observations $Y^{K}=\left\{M^{K},R^{K},S^{K},N^{K}\right\}$, we wish to estimate *x*_*k*_ ∀*k* and determine the set of unknown model parameters. We perform this using Bayesian filtering applied within an EM framework. At the E-step, we use both a forward filter and a backward smoother to estimate *x*_*k*_. At the M-step, we use the estimated values of *x*_*k*_ to obtain the next set of model parameters that maximizes the expected value of the complete data log likelihood. The algorithm iterates between the E-step and the M-step until convergence. We derive the forward filter equations and the M-step updates in S1 Appendix based on [43].

#### Expectation step

We make a Gaussian approximation to the posterior density *p*(*x*_*k*_|*y*^*k*^) similar to [43] to obtain the following filter equations for *k* = 2: *K*.

*Predict*:

$$\begin{array}{cc} x_{k|k-1} & =\rho x_{k-1|k-1}+\alpha I_{k} \end{array}$$ (12)

$$\begin{array}{cc} \sigma_{k|k-1}^{2} & =\rho^{2}\sigma_{k-1|k-1}^{2}+\sigma_{\varepsilon }^{2} \end{array}$$ (13)

*Update*:

$$\begin{array}{cc} c_{k} & =\frac{\sigma_{k|k-1}^{2}}{\sigma_{v}^{2}\sigma_{w}^{2}+\sigma_{k|k-1}^{2}\left(\gamma_{1}^{2}\sigma_{w}^{2}+\delta_{1}^{2}\sigma_{v}^{2}\right)} \end{array}$$ (14)

$$\begin{array}{cc} x_{k|k} & =x_{k|k-1}+c_{k}[\beta_{1}\sigma_{v}^{2}\sigma_{w}^{2}\left(m_{k}-p_{k|k}\right)+\gamma_{1}\sigma_{w}^{2}\left(r_{k}-\gamma_{0}-\gamma_{1}x_{k|k-1}\right)\\ & \;+\delta_{1}\sigma_{v}^{2}\left(s_{k}-\delta_{0}-\delta_{1}x_{k|k-1}\right)+\sigma_{v}^{2}\sigma_{w}^{2}\sum_{j=1}^{J}\frac{1}{\lambda_{k,j|k}}\frac{\partial \lambda_{k,j|k}}{\partial x_{k}}\left(n_{k,j}-\lambda_{k,j|k}\Delta \right)] \end{array}$$ (15)

$$\begin{array}{cc} \sigma_{k|k}^{2} & =\{\frac{1}{\sigma_{k|k-1}^{2}}+\beta_{1}^{2}p_{k|k}\left(1-p_{k|k}\right)+\frac{\gamma_{1}^{2}}{\sigma_{v}^{2}}+\frac{\delta_{1}^{2}}{\sigma_{w}^{2}}-\sum_{j=1}^{J}[\frac{1}{\lambda_{k,j|k}}\frac{\partial^{2}\lambda_{k,j|k}}{\partial x_{k}^{2}}\left(n_{k,j}-\lambda_{k,j|k}\Delta \right)\\ & \;-\frac{n_{k,j}}{\lambda_{k,j|k}^{2}}{\left(\frac{\partial \lambda_{k,j|k}}{\partial x_{k}}\right)}^{2}]{\}}^{-1} \end{array}$$ (16)

These equations are similar to the Kalman filter predict and update steps. Eq (15) applies a correction to *x*_*k*|*k*−1_ based on a comparison between the skin conductance and heart rate measurements observed at time index *k* and their model predictions. For instance, the phasic-derived *r*_*k*_ is compared to its model prediction *γ*_0_ + *γ*_1_ *x*_*k*|*k*−1_ and the appearance of an SCR *m*_*k*_ is compared to its predicted probability *p*_*k*|*k*_. Eq (15) is also solved numerically using Newton’s method as *x*_*k*|*k*_ appears on both sides of the equality sign [51]. The smoothed state and variance estimates *x*_*k*|*K*_ and $\sigma_{k|K}^{2}$ are [80]

$$\begin{array}{cc} A_{k} & ≜\rho \frac{\sigma_{k|k}^{2}}{\sigma_{k+1|k}^{2}} \end{array}$$ (17)

$$\begin{array}{cc} x_{k|K} & =x_{k|k}+A_{k}\left(x_{k+1|K}-x_{k+1|k}\right) \end{array}$$ (18)

$$\begin{array}{cc} \sigma_{k|K}^{2} & =\sigma_{k|k}^{2}+A_{k}^{2}\left(\sigma_{k+1|K}^{2}-\sigma_{k+1|k}^{2}\right). \end{array}$$ (19)

#### Maximization step—Model parameters related to skin conductance

The model parameters $\rho ,\alpha ,\beta_{0},\beta_{1},\delta_{0},\delta_{1},\sigma_{w}^{2},\gamma_{0},\gamma_{1},\sigma_{v}^{2}$ and $\sigma_{\varepsilon }^{2}$ are calculated at the M-step. Making use of the state-space covariances in [81] and defining the following

$$\begin{array}{cc} U_{k} & ≜x_{k|K}^{2}+\sigma_{k|K}^{2} \end{array}$$ (20)

$$\begin{array}{cc} U_{k,k+1} & ≜x_{k|K}x_{k+1|K}+A_{k}\sigma_{k+1|K}^{2}, \end{array}$$ (21)

we obtain the following updates for the (*n* + 1)^th^ EM iteration.

$$\begin{array}{cc} {}[\begin{array}{c} \rho^{\left(n+1\right)}\\ \alpha^{\left(n+1\right)} \end{array}]= & {\left[\begin{array}{cc} \sum_{k=1}^{K-1}U_{k} & \sum_{k=2}^{K}I_{k}x_{k-1|K}\\ \sum_{k=2}^{K}I_{k}x_{k-1|K} & \sum_{k=1}^{K}I_{k}^{2} \end{array}\right]}^{-1}[\begin{array}{c} \sum_{k=1}^{K-1}U_{k,k+1}\\ \sum_{k=2}^{K}I_{k}x_{k|K} \end{array}] \end{array}$$ (22)

$$\begin{array}{cc} {}[\begin{array}{c} \gamma_{0}^{\left(n+1\right)}\\ \gamma_{1}^{\left(n+1\right)} \end{array}]= & {\left[\begin{array}{cc} K & \sum_{k=1}^{K}x_{k|K}\\ \sum_{k=1}^{K}x_{k|K} & \sum_{k=1}^{K}U_{k} \end{array}\right]}^{-1}[\begin{array}{c} \sum_{k=1}^{K}r_{k}\\ \sum_{k=1}^{K}r_{k}x_{k|K} \end{array}] \end{array}$$ (23)

$$\begin{array}{cc} \sigma_{v}^{2(n+1)}= & \frac{1}{K}[\sum_{k=1}^{K}r_{k}^{2}+K\gamma_{0}^{2(n+1)}+\gamma_{1}^{2(n+1)}\sum_{k=1}^{K}U_{k}\\ & -2\gamma_{0}^{\left(n+1\right)}\sum_{k=1}^{K}r_{k}-2\gamma_{1}^{\left(n+1\right)}\sum_{k=1}^{K}x_{k|K}r_{k}+2\gamma_{0}^{\left(n+1\right)}\gamma_{1}^{\left(n+1\right)}\sum_{k=1}^{K}x_{k|K}] \end{array}$$ (24)

$$\begin{array}{cc} \sigma_{\varepsilon }^{2(n+1)}= & \frac{1}{K}[\sum_{k=2}^{K}U_{k}-2\rho^{\left(n+1\right)}\sum_{k=1}^{K-1}U_{k,k+1}+\rho^{2(n+1)}\sum_{k=1}^{K-1}U_{k}-2\alpha^{\left(n+1\right)}\sum_{k=2}^{K}I_{k}x_{k|K}\\ & +2\alpha^{\left(n+1\right)}\rho^{\left(n+1\right)}\sum_{k=2}^{K}I_{k}x_{k-1|K}+\alpha^{2(n+1)}\sum_{k=1}^{K}I_{k}^{2}] \end{array}$$ (25)

The M-step updates are likewise obtained for *δ*_0_ and *δ*_1_ by replacing *r*_*k*_ with *s*_*k*_ in Eq (23). The update for $\sigma_{w}^{2}$ can be obtained similarly by making the corresponding changes to *γ*_0_, *γ*_1_ and *r*_*k*_ in Eq (24).

Estimating *β*_0_ and *β*_1_ requires the maximization of (S1 Appendix) [43]

$$\begin{array}{c} {\bar{Q}}_{1}=\sum_{k=1}^{K}E[m_{k}\left(\beta_{0}+\beta_{1}x_{k}\right)-\log(1+e^{\beta_{0}+\beta_{1}x_{k}})]. \end{array}$$ (26)

Owing to the difficulty of analytically computing ${\bar{Q}}_{1}$, two alternate approaches (based on approximations) are commonly used in the literature for estimating *β*_0_ and *β*_1_. These are as follows:

1. The first approach is to set *β*_1_ = 1 and calculate *β*_0_ empirically [51, 52]. This results from using the model
  $$\begin{array}{cc} \log(\frac{p_{k}}{1-p_{k}})=\beta_{0}+x_{k} & \Rightarrow p_{k}=\frac{1}{1+e^{-(\beta_{0}+x_{k})}}. \end{array}$$ (27)
  It can be assumed that *x*_*k*_ ≈ 0 at the very beginning. Therefore
  $$\begin{array}{c} \log(\frac{p_{0}}{1-p_{0}})\approx \beta_{0}. \end{array}$$ (28)
  *β*_0_ can now be calculated from Eq (28) taking *p*_0_ as the chance probability that *m*_*k*_ = 1. For instance, in behavioral learning experiments involving correct/incorrect responses, *p*_0_ is the probability that a subject gets an answer correct at the very outset prior to any learning occurring (i.e., the chance probability) [51, 52]. In our prior work on Bayesian filtering using skin conductance data, taking *p*_0_ as the average probability of an SCR occurring in the whole experiment provided good results [42, 45, 82, 83].
2. The second approach is by means of a Taylor series expansion [43]. Each of the summed terms in ${\bar{Q}}_{1}$ can be expanded around *x*_*k*|*K*_. Thereafter, ${\bar{Q}}_{1}$ can be approximated by only using the first few Taylor series terms. The derivatives of the approximated ${\bar{Q}}_{1}$ with respect to *β*_0_ and *β*_1_ can then be taken and set to zero to find the M-step updates. These derivatives yield the following (S1 Appendix) [43]:
  $$\begin{array}{cc} \sum_{k=1}^{K}[m_{k}-p_{k|K}-\frac{1}{2}\beta_{1}^{2(n+1)}\sigma_{k|K}^{2}p_{k|K}\left(1-p_{k|K}\right)\left(1-2p_{k|K}\right)] & \approx 0 \end{array}$$ (29)
  $$\begin{array}{cc} \sum_{k=1}^{K}\{m_{k}x_{k|K}-x_{k|K}p_{k|K}-\frac{1}{2}\beta_{1}^{\left(n+1\right)}\sigma_{k|K}^{2}p_{k|K}\left(1-p_{k|K}\right)\\ \left[2+\beta_{1}^{\left(n+1\right)}x_{k|K}\left(1-2p_{k|K}\right)]\right\} & \approx 0. \end{array}$$ (30)
  These two equations can be numerically solved using MATLAB’s *fsolve* function to provide the M-step updates for *β*_0_ and *β*_1_.

Both types approximations described above provide reasonably good results in our own experience and either option can be used. Note that the *β*_0_ and *β*_1_ coefficients appear in exponents, as in Eq (2) for instance, and state estimation can be sensitive to them. Due to this sensitivity in the exponent terms, the second approximation option can cause convergence issues as it tries to iteratively estimate *β*_0_ and *β*_1_ at the M-step. In contrast, *β*_0_ and *β*_1_ are calculated using alternate means in the first option. Thus it is less likely to have difficulty converging. We use a convergence criteria similar to [52] and consider all model parameters estimated at the M-step to have converged once the mean absolute difference between successive iterations does not exceed a specified tolerance level. Here, we use a tolerance level in the order of 10^−5^–10^−6^ on simulated and experimental data.

#### Maximization step—Model parameters related to heart rate

Ideally, all model parameters related to both skin conductance and heart rate should be estimated at the M-step simultaneously. Recall that we have to determine *θ*_0_, *θ*_1_, …, *θ*_*q*+1_ and *η* for heart rate. Calculating these values at the M-step requires the maximization of (S1 Appendix) [43]

$$\begin{array}{cc} {\bar{Q}}_{2} & =\sum_{k=1}^{K}\sum_{j=1}^{J}E[\log\left(\lambda_{k,j}\Delta \right)n_{k,j}-\lambda_{k,j}\Delta ]. \end{array}$$ (31)

Maximizing ${\bar{Q}}_{2}$ with respect to the *θ*_*i*_’s and *η* for a fixed model order *q* is extremely time consuming. Additionally, multiple values of *q* need to be evaluated for selecting the best order. Owing to the large time complexity, we resorted to an alternate two-step strategy for determining the model parameters related to heart rate.

**Fig 5:**
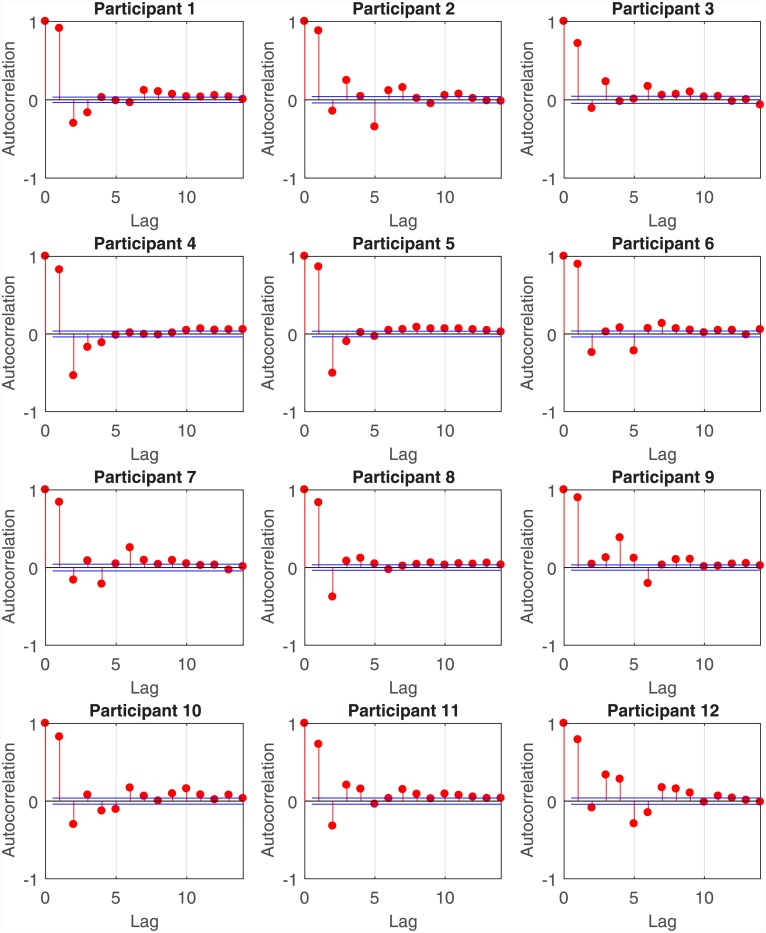
Partial autocorrelation analysis of RR-intervals. For all participants, the partial autocorrelation values are large at the first few lags and then become smaller. For many participants, the dependence of the current RR-interval on a lag of beyond eight is small. Consequently, we chose to investigate model orders *q* = 1, 2, …, 8 for each participant and selected the value of *q* giving rise to the smallest KS distance.

- Step 1: Determining the model order *q* and the *θ*_*i*_ coefficients
  The HDIG density function models the RR-interval mean as a weighted sum of the previous *q* RR-intervals. This is similar to an AR model where a value in a time series is predicted from its past values [78]. Barbieri et al. [73] used the HDIG density function to model RR-intervals in ten subjects who participated in a tilt-table experiment. They investigated different model orders *q* and selected *q* = 2 for two subjects and *q* = 4 for eight subjects based on goodness-of-fit and Akaike’s information criteria. Goodness-of-fit was assessed using the Kolmogorov-Smirnov (KS) plot. The KS plot is based on the time rescaling theorem [84] and provides an indication of how good a CIF fits to point process observations. The time rescaling theorem is frequently used in the analysis of neural spike trains [85–87] and heartbeats [73, 75, 78, 88]. The closer the KS plot is to the 45°diagonal, the better the fit is to the observed binary point process data. Thus, the maximum distance between the KS plot and the 45°diagonal (known as the KS distance), provides a quantitative measure of goodness-of-fit. In [78], Barbieri et al. performed a partial autocorrelation analysis of RR-intervals and selected an AR(8) HDIG model order for a tilt table study. Similar to [78], we too perform a partial autocorrelation analysis of the subjects’ RR-intervals (Fig 5). For many subjects, the autocorrelation terms beyond a lag of eight tend to be small in comparison to the first few lags. We therefore chose to investigate model orders up to *q* = 8. For each model order *q*, we estimated *θ*_0_, *θ*_1_, …, *θ*_*q*+1_ offline via maximum likelihood [73]. The best model order *q* and the *θ*_*i*_ coefficients were selected based on the smallest KS distance (Table 1). We performed step 1 for each participant prior to arousal estimation using EM.
- Step 2: Determining *η*
  After selecting the *θ*_*i*_’s and the model order *q*, we run the full EM algorithm for arousal estimation for a fixed set of values for *η*. Since RR-intervals would decrease (i.e., heart rate would speed up) with increased sympathetic drive, we chose to try the set of negative values {−10^−6^, −10^−5^, −10^−4^, −10^−3^, −10^−2^, −10^−1^} for *η*. We resorted to this two-step strategy because determining *η* and the *θ*_*i*_’s simultaneously at the M-step proved to be extremely time consuming. ${\bar{Q}}_{2}$ in Eq (31) can be approximated by
  $$\begin{array}{cc} {\bar{Q}}_{2} & \approx \sum_{k=1}^{K}\sum_{j=1}^{J}\log\left(\lambda_{k,j|K}\Delta \right)n_{k,j}-\lambda_{k,j|K}\Delta +\frac{1}{2}[\frac{1}{\lambda_{k,j|K}}\frac{\partial^{2}\lambda_{k,j|K}}{\partial x_{k}^{2}}\left(n_{k,j}-\lambda_{k,j|K}\Delta \right)\\ & \;-\frac{n_{k,j}}{\lambda_{k,j|K}^{2}}{\left(\frac{\partial \lambda_{k,j|K}}{\partial x_{k}}\right)}^{2}]\sigma_{k|K}^{2}. \end{array}$$ (32)
  We derive this in S1 Appendix. Ideally, the *η* value with the largest ${\bar{Q}}_{2}$ during state estimation should be selected. However, the inclusion of *x*_*k*_ in the HDIG model causes the KS plot obtained via maximum likelihood to change. Therefore, *η* should be chosen to maximize ${\bar{Q}}_{2}$ subject to the new KS plot falling within or reasonably close to the 95% confidence bounds to the 45°diagonal (based on the time rescaling theorem, the 45°diagonal corresponds to the perfect CIF estimate for a given set of point process observations).

**Table 1:** Model parameter selection for heart rate.

Original study subject number	Participant	Model order *q*	KS distance
5	1	3	0.021327
6	2	8	0.025880
7	3	1	0.019057
10	4	6	0.063215
11	5	6	0.047393
12	6	1	0.022211
13	7	3	0.015398
14	8	2	0.030028
16	9	5	0.022618
17	10	2	0.028208
18	11	8	0.016154
19	12	4	0.019527

KS plots are frequently used to evaluate the goodness-of-fit to spiking-type observations (e.g. single neuron spiking, R-peaks). Here we selected *q* and the *θ*_*i*_ coefficients based on the minimum KS distances. The subject ID numbers according to the original study are also shown in the table.

## Results

### Simulated data

We simulated two sets of data to check the ability of our model to estimate an unobserved arousal state and recover model parameters. The model parameters were chosen based on prior experience with skin conductance and heart rate data. We used the first approximation strategy for *β*_0_ and *β*_1_ (i.e., setting *β*_1_ = 1 and calculating *β*_0_ based on an empirical approximation). We used *p*_0_ = 0.01 to generate *β*_0_ (i.e., *β*_0_ = log[*p*_0_(1 − *p*_0_)^−1^]). Recall that during estimation, we calculate *β*_0_ empirically by estimating ${\hat{p}}_{0}$ as the average probability that *m*_*k*_ = 1 in the data. The two simulated data sets correspond to two instances where ${\hat{p}}_{0}>0.01$ and ${\hat{p}}_{0}<0.01$. We also set the indicator function *I*_*k*_ = 1 at 25 arbitrary locations.

**Fig 6:**
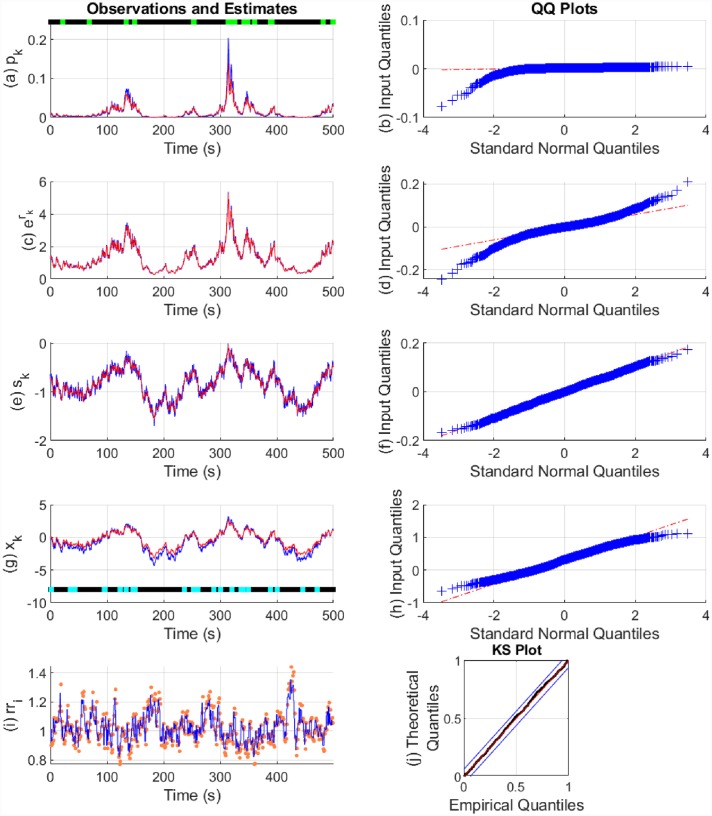
State estimation with simulated data (${\hat{p}}_{0}>0.01$). The sub-panels respectively depict, (a) the Bernoulli trial probabilities *p*_*k*_ (blue), their estimate (red) and the presence or absence of binary observations (light green and black dots); (b) the quantile-quantile (QQ) plot for the residual error of *p*_*k*_; (c) the exponent of *r*_*k*_ (blue) and its estimate (red); (d) the QQ plot for the residual error of *r*_*k*_; (e) *s*_*k*_ (blue) and its estimate (red); (f) the QQ plot for the residual error of *s*_*k*_; (g) the arousal state *x*_*k*_ (blue), its estimate (red) and the presence or absence of stimuli *I*_*k*_ driving the state (cyan and blacks dots); (h) the QQ plot for the residual error of *x*_*k*_; (i) the sequence of RR-intervals rr_i_ (orange dots) and the estimated RR-interval mean *μ* (solid blue line); (j) the KS plot for the heartbeats.

**Fig 7:**
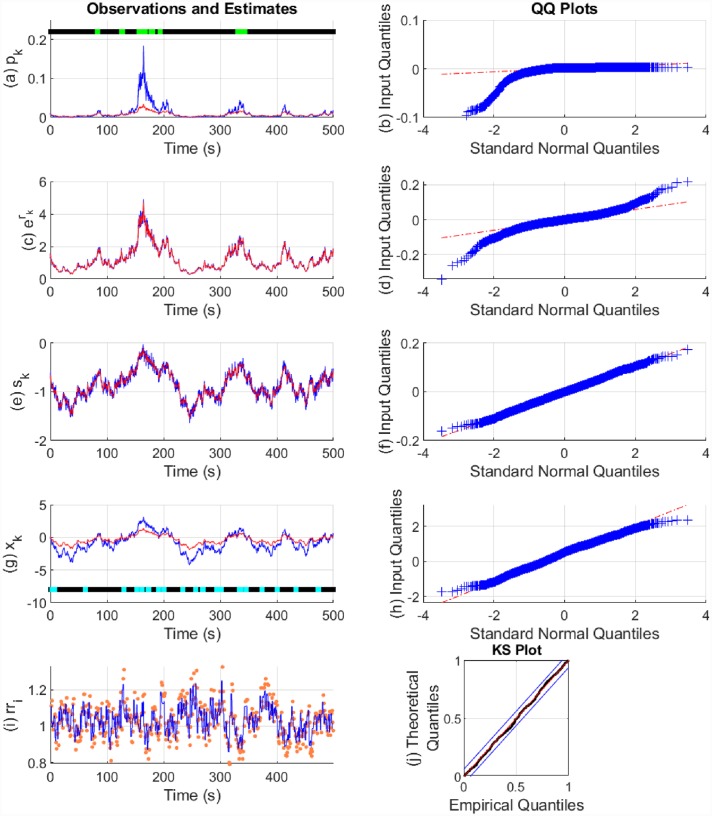
State estimation with simulated data (${\hat{p}}_{0}<0.01$). The sub-panels respectively depict, (a) the Bernoulli trial probabilities *p*_*k*_ (blue), their estimate (red) and the presence or absence of binary observations (light green and black dots); (b) the QQ plot for the residual error of *p*_*k*_; (c) the exponent of *r*_*k*_ (blue) and its estimate (red); (d) the QQ plot for the residual error of *r*_*k*_; (e) *s*_*k*_ (blue) and its estimate (red); (f) the QQ plot for the residual error of *s*_*k*_; (g) the arousal state *x*_*k*_ (blue), its estimate (red) and the presence or absence of stimuli *I*_*k*_ driving the state (cyan and blacks dots); (h) the QQ plot for the residual error of *x*_*k*_; (i) the sequence of RR-intervals rr_i_ (orange dots) and the estimated RR-interval mean *μ* (solid blue line); (j) the KS plot for the heartbeats.

The state estimation results are shown in Figs 6 and 7. The model parameters used and their estimates are shown in Table 2. In both cases, there is a good fit to the continuous-valued observations *r*_*k*_ and *s*_*k*_. In the case of ${\hat{p}}_{0}>0.01$, the fits are better to the binary observations and to the states. The approximation with ${\hat{p}}_{0}<0.01$ tends to underestimate the probability of the binary observations. This is likely because *β*_0_ and *β*_1_ appear in the exponents, and estimation is therefore more sensitive to them. The fits to the heartbeats are also good with the KS plot lying within the 95% confidence bounds. We calculated the *θ*_*i*_ coefficients separately using maximum likelihood and estimated *η* from a fixed set of values using the EM algorithm.

**Table 2:** Parameter estimation with simulated data.

Parameter value	Estimated value (${\hat{p}}_{0}>0.01$)	Estimated value (${\hat{p}}_{0}<0.01$)
*α* = 0.04	0.0082	0.0281
*ρ* = 0.995	0.9941	0.9947
*δ*_0_ = −0.7	-0.739	-0.704
*δ*_1_ = 0.2	0.2532	0.4417
$\sigma_{w}^{2}=0.003$	0.003	0.0031
*γ*_0_ = 0.35	0.2716	0.3422
*γ*_1_ = 0.4	0.5057	0.8848
$\sigma_{v}^{2}=0.002$	0.0019	0.0022
*β*_0_ = −4.5951	-4.5458	-4.7015
*β*_1_ = 1	1 (set)	1 (set)
$\sigma_{\varepsilon }^{2}=0.03$	0.0188	0.0058
*θ*_0_ = 0.27432	0.24028	0.29978
*θ*_1_ = 0.83697	0.83787	0.76209
*θ*_2_ = −0.10511	-0.07417	-0.05446
*θ*_3_ = 234.22144	239.28030	237.89351
*η* = −0.005	-0.001	-0.001

We simulated two sets of data with the same set of parameters. For one of them, the approximation for *p*_0_ was slightly less than the true value and for the other it was slightly above.

### Experimental data

We set *I*_*k*_ = 1 corresponding to the times at which the CS+, CS- and US stimuli were presented. Unlike in the case of simulated data, additional constraints had to be placed when running the EM algorithm on experimental data. Here, there was a tendency for the model parameters to converge to a location where there was an almost perfect fit to one of the continuous-valued observations (either *r*_*k*_ or *s*_*k*_). It is likely that local extrema exist in the model parameter search space at these points and the EM algorithm can converge to them. In order to avoid *x*_*k*_ overfitting to *r*_*k*_ or to *s*_*k*_, we first divided them by their respective standard deviations and then monitored the variance terms $\sigma_{v}^{2(n+1)}$ and $\sigma_{w}^{2(n+1)}$ at each iteration. All the model parameters were only allowed to update if the absolute difference between the variance terms exceeded 0.1. Thus the EM algorithm was prevented from overfitting by driving down one of the variance terms at the expense of the other. The EM iterations were stopped once it was detected that overfitting (as measured by the variance difference criteria) to either *r*_*k*_ or *s*_*k*_ would occur at the next iteration. This approach is similar to the early stopping criteria used to prevent overfitting when training artificial neural networks via gradient descent [89]. We also calculated *β*_1_ and *β*_2_ at the M-step as overfitting, rather than convergence, is the major concern with experimental data. We also included an additional constraint to prevent the *α* coefficient from becoming negative during estimation as this would imply that the external stimuli (for instance, the electric shock) decreases arousal. The model parameter estimates are given in S1 Appendix.

**Fig 8:**
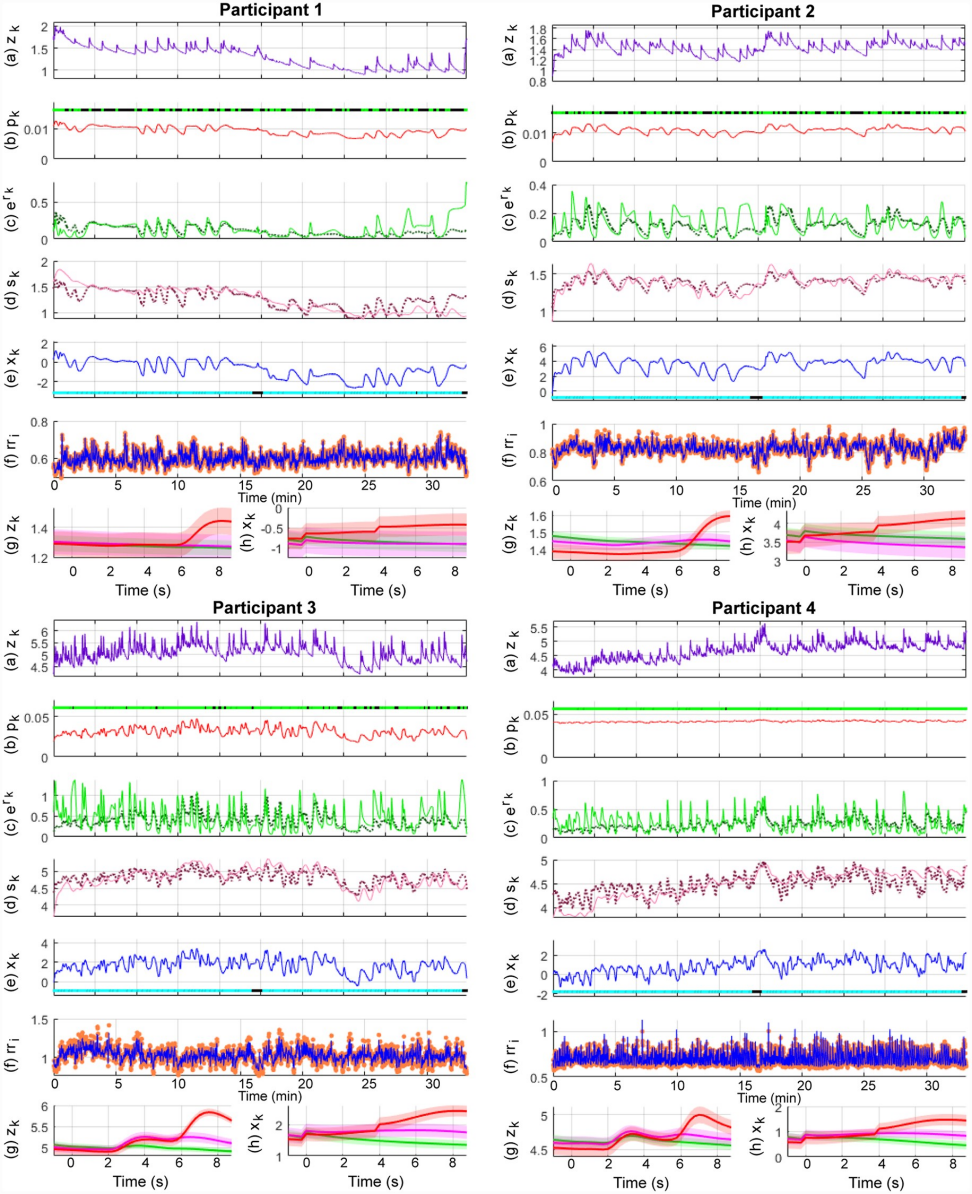
Sympathetic arousal estimation for participants 1-4 in the trace fear conditioning experiment. The sub-panels from top to bottom respectively depict, (a) the skin conductance signal *z*_*k*_; (b) the presence or absence of SCR peaks (light green and black dots) and the smoothed Bernoulli trial probability estimates of *p*_*k*_ (red line); (c) the exponent of the phasic derived signal *r*_*k*_ (solid green line) and its estimate (dotted line); (d) the tonic part *s*_*k*_ (solid light mauve line) and its estimate (dotted line); (e) the smoothed arousal state estimates of *x*_*k*_ and the presence or absence of visual or electric stimuli (cyan and blacks dots); (f) the sequence of RR-intervals rr_i_ (orange dots) and the estimated RR-interval mean *μ* (solid blue line); (g) a 10 s ERP-like skin conductance plot for the CS- (green), CS+ without a shock (mauve—CS+US-) and CS+ with the shock (red—CS+US+) trials; (h) 10 s ERP-like arousal state plots along with their confidence intervals.

**Fig 9:**
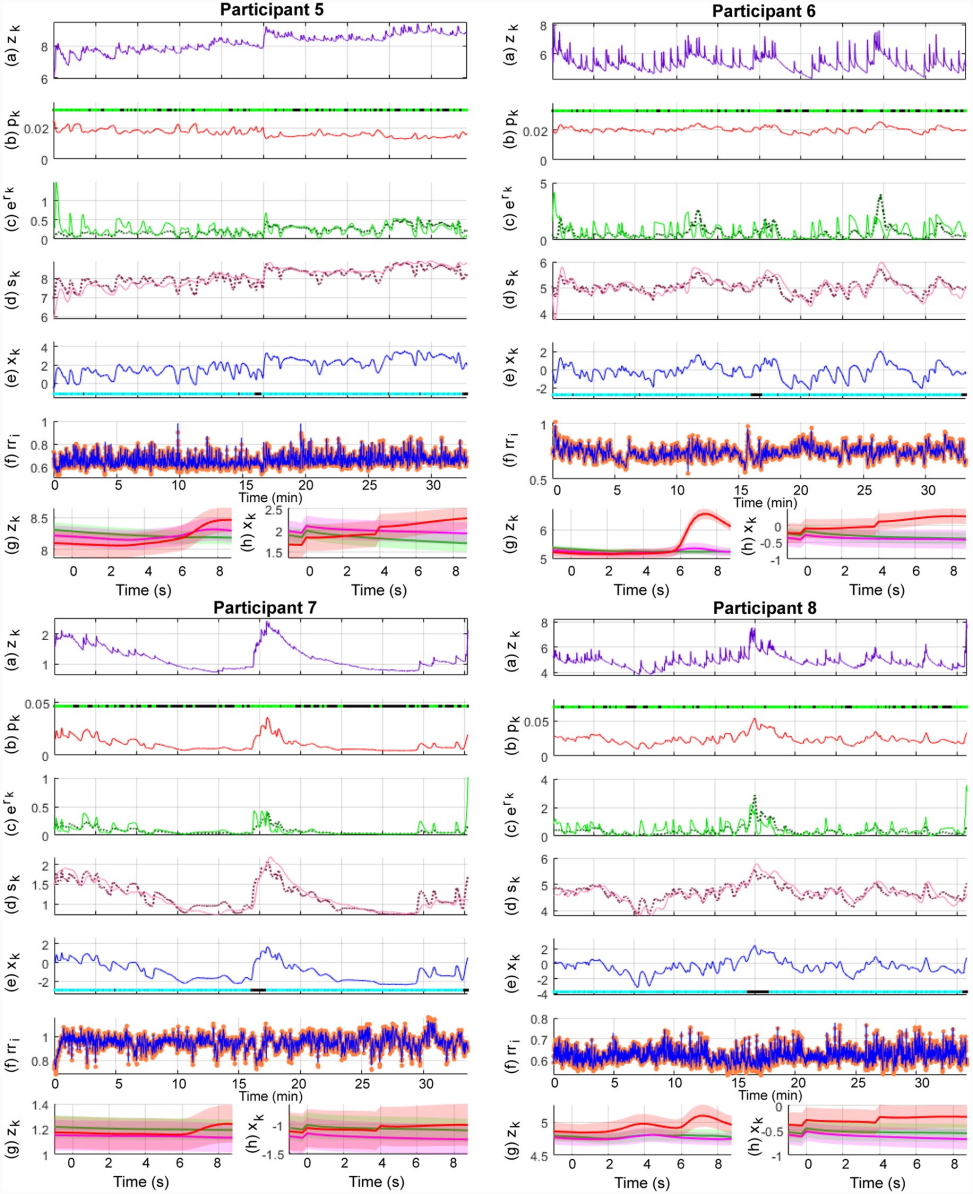
Sympathetic arousal estimation for participants 5-8 in the trace fear conditioning experiment. The sub-panels from top to bottom respectively depict, (a) the skin conductance signal *z*_*k*_; (b) the presence or absence of SCR peaks (light green and black dots) and the smoothed Bernoulli trial probability estimates of *p*_*k*_ (red line); (c) the exponent of the phasic derived signal *r*_*k*_ (solid green line) and its estimate (dotted line); (d) the tonic part *s*_*k*_ (solid light mauve line) and its estimate (dotted line); (e) the smoothed arousal state estimates of *x*_*k*_ and the presence or absence of visual or electric stimuli (cyan and blacks dots); (f) the sequence of RR-intervals rr_i_ (orange dots) and the estimated RR-interval mean *μ* (solid blue line); (g) a 10 s ERP-like skin conductance plot for the CS- (green), CS+ without a shock (mauve—CS+US-) and CS+ with the shock (red—CS+US+) trials; (h) 10 s ERP-like arousal state plots along with their confidence intervals.

**Fig 10:**
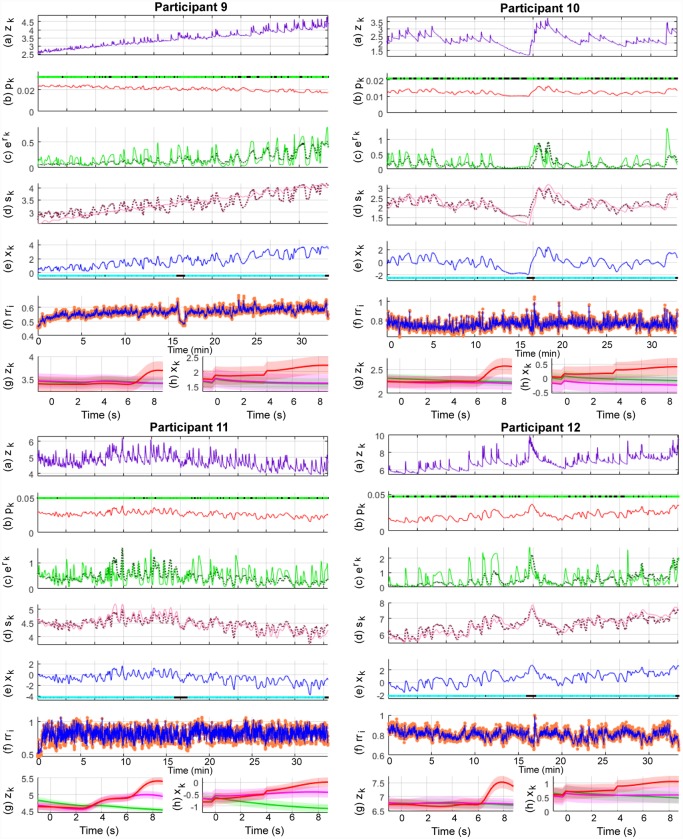
Sympathetic arousal estimation for participants 9-12 in the trace fear conditioning experiment. The sub-panels from top to bottom respectively depict, (a) the skin conductance signal *z*_*k*_; (b) the presence or absence of SCR peaks (light green and black dots) and the smoothed Bernoulli trial probability estimates of *p*_*k*_ (red line); (c) the exponent of the phasic derived signal *r*_*k*_ (solid green line) and its estimate (dotted line); (d) the tonic part *s*_*k*_ (solid light mauve line) and its estimate (dotted line); (e) the smoothed arousal state estimates of *x*_*k*_ and the presence or absence of visual or electric stimuli (cyan and blacks dots); (f) the sequence of RR-intervals rr_i_ (orange dots) and the estimated RR-interval mean *μ* (solid blue line); (g) a 10 s ERP-like skin conductance plot for the CS- (green), CS+ without a shock (mauve—CS+US-) and CS+ with the shock (red—CS+US+) trials; (h) 10 s ERP-like arousal state plots along with their confidence intervals.

**Fig 11:**
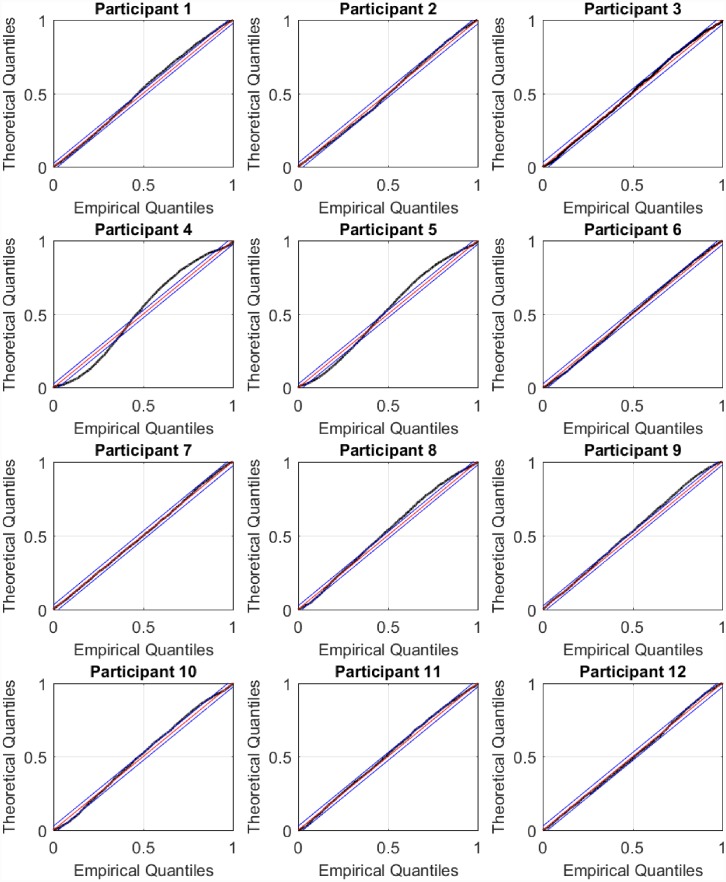
KS plots for the participants. The KS plots for the participants lie close to the 45°diagonal indicating a good fit to the heartbeats (a point process). Deviations from the 45°diagonal are most prominent for participants 4 and 5.

Pavlovian fear conditioning experiments have often sought to examine average differences in physiological features between the trial types. Since this is similar to the study of event-related potentials (ERPs) with EEG data, we provide the ERP-like images for the three types of trials—CS-, CS+ without the US (CS+US-) and CS+ with the US (CS+US+). The state estimation results are shown in Figs 8, 9 and 10 and the KS plots for fits to the heartbeats are shown in Fig 11. The mean and standard deviation of *x*_*k*_ within each of the 10 s periods considered for the ERP-like plots is shown in Table 3.

**Table 3:** Mean and standard deviation of *x*_*k*_ in different trial conditions.

Category	Participant	CS+US+		CS+US-		CS-		CS+US+ > CS+US-	CS+US- > CS-
		mean	s.d.	mean	s.d.	mean	s.d.	(*p*-value)	(*p*-value)
A	3	1.9720	0.3203	1.7754	0.0499	1.5035	0.1138	0.0001*	< 0.0001*
	4	1.0546	0.3222	0.8771	0.0687	0.6756	0.1183	0.0005*	< 0.0001*
	5	1.9982	0.1939	1.9864	0.0427	1.8153	0.0794	0.3537	< 0.0001*
	11	-0.4086	0.2796	-0.5008	0.0904	-0.9051	0.1477	0.0253*	< 0.0001*
Overall (A)		1.1541	1.0215	1.0345	0.9846	0.7723	1.0639		
B	1	-0.5490	0.1140	-0.8844	0.0248	-0.8402	0.0493	< 0.0001*	> 0.9999
	6	0.0515	0.1725	-0.3859	0.0321	-0.3178	0.0617	< 0.0001*	> 0.9999
	9	1.9902	0.1579	1.6591	0.0429	1.6319	0.0428	< 0.0001*	0.0029*
	12	0.8392	0.1611	0.5776	0.0224	0.5804	0.0628	< 0.0001*	0.6034
Overall (B)		0.5830	0.9649	0.2416	0.9762	0.2636	0.9438		
C	7	-1.0530	0.0416	-1.2204	0.0276	-1.0636	0.0220	< 0.0001*	> 0.9999
	8	-0.2820	0.0568	-0.6205	0.0398	-0.5316	0.0235	< 0.0001*	> 0.9999
	10	0.2368	0.1206	-0.2129	0.0382	-0.0371	0.0433	< 0.0001*	> 0.9999
Overall (C)		-0.3661	0.5381	-0.6846	0.4170	-0.5441	0.4221		
–	2	3.8671	0.2012	3.4755	0.0753	3.6719	0.0546	<0.0001*	> 0.9999
Overall		0.8098	1.3836	0.5438	1.3742	0.5153	1.3683		

Fear conditioning experiments frequently examine physiological responses across the different types of trials. The table shows the mean and standard deviation of the averaged *x*_*k*_ values over the 10 s period shown in Figs 8, 9 and 10 for the CS+US+, CS+US- and CS- trials. The results for the participants are shown according to the categories A, B and C, and *p*-values less than 0.05 are indicated with a *.

We first consider Figs 8, 9 and 10. Overall, both the skin conductance and estimated arousal states are highest in the CS+US+ trials. The participants may be divided into three categories based on their physiological responses and state estimates. Participants 3, 4, 5 and 11 have very similar averaged responses. We group them in category A. For each of the participants in category A, the average response to the CS+US+ is highest followed by the CS+US-. The CS- trials have the lowest average response. This is as expected. Clear gaps are visible between the averaged responses for each of the three types of trials. The gaps are visible for both averaged skin conductance and arousal. In category B are participants 1, 6, 9 and 12. For these participants, the gap between the CS+US- and CS- trials is very small. However, the average response for the CS+US+ is still larger. For participants 1 and 9, the averaged CS+US- and CS- curves lie almost on top of each other. For participant 6, there is a small rise in the averaged CS+US- skin conductance curve above the CS- curve while the corresponding state estimates are very close to each other. There is however, a slight upward trend in the averaged CS+US- arousal state curve curve and a corresponding downward trend in the averaged CS- curve. Participants 7, 8 and 10 are grouped into category C. Here, the general trend is that the averaged CS- curve tends to exceed the CS+US- curve. Participant 2 is an exception. Here, the averaged skin conductance and state estimates for the CS- and CS+US- are interchanged. We provide a discussion of these results in the following section. We include a separate discussion for participant 2 for whom there appears to be a mismatch between the averaged skin conductance and arousal state estimate curves. Participant 2 appears to develop a skin conductance arousal response to the CS- trials towards the end of the experiment. This is unusual as the participant should have by then learned that the CS- trials are never accompanied by the electric shock.

We next consider Table 3 which summarizes the results from the ERP-like plots in the figures. Again, consistent with Figs 8, 9 and 10, the highest *x*_*k*_ values generally occur in the CS+US+ trials. The difference between the CS+US- and CS- trials is less distinguishable and there are differences between participants. We have grouped the participants into categories A, B and C in the table. The means and standard deviations of the arousal state *x*_*k*_ for each category are also shown here. The responses for individual participants are generally as expected in category A. For individual participants in categories B and C, the responses are not as would be expected in a typical fear conditioning experiment since the mean values for the CS- trials sometimes exceed those of the CS+US- trials. For category A as a whole, the mean value for *x*_*k*_ is largest in the CS+US+ trials followed by the CS+US- trials and then by the CS- trials. For category B, the mean value for the CS- trials is larger than that for the CS+US- trials. It is the same for category C, but the difference is larger. We also performed one-tailed t-tests to check if the means for CS+US+ trials were greater than those of the CS+US- trials, and if the means of the CS+US- trials were larger than those of the CS- trials for each participant. In general, the differences are significant in both cases for participants in category A. The differences are less apparent for participants in categories B and C. The weaker response to the CS+US- trials may be in part due to the use of the trace, rather than the delay, fear conditioning paradigm as we describe in the subsequent section. Other possibilities include an insufficient unpleasantness of the US on a per-subject level.

The KS plots for the participants are close to the 45°diagonal indicating a good fit to the heartbeat observations. Deviations outside the 95% confidence bounds are most prominent for participants 4 and 5. The HDIG model developed by Barbieri et al. [73, 75] uses time-varying *θ*_*i*_ coefficients that are estimated every 5 ms. The use of a fixed set of *θ*_*i*_’s estimated via maximum likelihood together with changes in arousal may have been insufficient to account for the HRV stochasticity completely. We have discussed how time-varying HDIG parameters may be incorporated into the model in the following section.

## Discussion

### Simulated data

The presence of binary-valued observations requires the use of a data transformation according to the theory of generalized linear models. Here, we use the logit transform to relate *m*_*k*_ = {0, 1} to *x*_*k*_ similar to [51, 52]. The logit transform necessitates the estimation of two parameters that appear in exponents (*β*_0_ and *β*_1_). Estimating exponents can be challenging as a small change may have a significant effect. We earlier noted two approaches found in the literature that are based on approximations which can be used to estimate *β*_0_ and *β*_1_. One of the approaches is less likely to cause difficulties in converging to parameters. We use this approach to test the ability of our model to estimate an unobserved arousal state and estimate model parameters. While we obtain good results with simulated data, the ability to fit to the binary observations was better in one case (${\hat{p}}_{0}>0.01$) than in the other. This is likely due to the sensitivity of the model to the exponent terms. The need to estimate exponent terms and the sensitivity thereof are limitations of the present model. An alternate strategy would be to use a different type of data transform on the binary data and relate it to *x*_*k*_. For instance, the complementary log-log and the inverse normal are additional transforms that are suggested for binary data [65]. These additional methods could be investigated in future to examine sensitivity to the exponents.

Numerical issues can also arise during state estimation. This is in part due to the use of the HDIG density function and its parameters. State estimation also depends on integrals and derivatives of the HDIG CIF over very small numbers. These factors can cause numerical issues during EM. A simpler probability density function or a Gaussian approximation to the HDIG density function may be helpful in avoiding some of the numerical instabilities that may arise. Yet another alternative would be to use particle filters with Monte-Carlo sampling for state estimation [90, 91].

### Experimental data

In Pavlovian fear conditioning, a neutral stimulus is paired with an unpleasant stimulus such as an electric shock. Through repeated exposure, a subject learns an association between the two types of stimuli and eventually begins to elicit a response typically associated with the unpleasant stimuli to the neutral predictor as well. Due to ethical considerations involved in causing pain to human subjects, the intensity of the electric shocks used in fear conditioning experiments is often adjusted to be “highly annoying, but not painful” [17]. In the data set used here, the shock intensity was adjusted to 90% of each subject’s pain threshold [28]. Subjects have different pain thresholds and an electric shock that is not painful enough may not cause the subject to fear the US as much. This is one of the possible reasons why variations are seen among the subjects analyzed in this data set. Ideally, we would expect to see the highest averaged responses (skin conductance and arousal) for CS+US+ and then for CS+US-. The CS- trials would be expected to have the lowest averaged responses. However, this clear difference is only visible in the four participants in category A. It appears that these four subjects learned the association between the CS+ cue and the electric shock and developed a fear response to the CS+ alone. In participants belonging to both the other categories, a clear separation with the averaged responses for CS+US- being higher than the averaged responses for CS- is not seen. There is almost no difference between the averaged responses for the CS+US- and CS- trials for participants in category B. The response is inverted for category C participants. The reason for responses such as those seen in category B and C is likely due to the participants not learning to fear the unpleasant electric shock enough. A further possibility for the lack of a response to the CS+ trials could be the type of experiment that was used. The data come from a trace fear conditioning experiment. In trace fear conditioning, there is a gap between the time the CS+ ends and the application of the US. In delay fear conditioning, the CS+ stimuli co-terminate with US without any time gap. Due to the closer pairing in time, the response to the CS+ stimuli in delay fear conditioning is usually larger than in trace fear conditioning [17]. Trace conditioning involves the hippocampus while delay conditioning predominantly involves the amygdala [17]. Finally, the experiment included the electric shock only in 50% of the CS+ trials. Therefore, a participant learns that not all CS+ trials precede a shock. The use of: (i) trace conditioning, (ii) CS+ only accompanying the US in 50% of the trials and (iii) shocks that may not have been unpleasant enough are possible reasons why only four participants had a response as expected.

**Fig 12:**
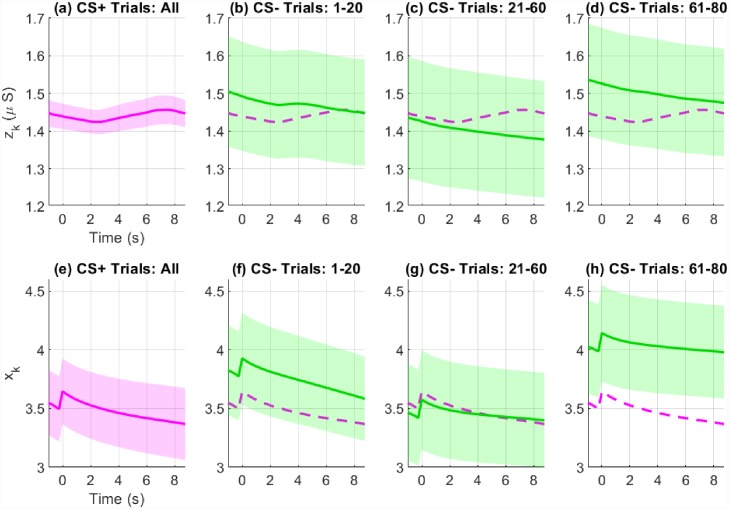
Variation of the averaged skin conductance and state estimates across trials for participant 2. Sub-panels (a) and (e) show the averaged skin conductance (*z*_*k*_) and arousal (*x*_*k*_) estimates for all CS+US- trials. Sub-panels (b)-(d) and (f)-(h) show the variation of the averaged skin conductance and arousal estimates for the CS- trials across trial blocks 1-20, 21-60 and 61-80. For *z*_*k*_, there is a drop from trials 1-20 to 21-60. However, there is an increase from 61-80. The same pattern is visible for *x*_*k*_. However, the gap is much larger for *x*_*k*_ in trials 61-80. Therefore when averaged across all blocks, the relative positions of the CS+US- and CS- are interchanged (Fig 8).

Participant 2 was a notable exception as the averaged skin conductance and state estimates for CS- and CS+US- do not match each other. A total of 160 trials were included in the fear conditioning experiment—40 CS+US+, 40 CS+US- and 80 CS- trials. The trials occur in random order. Fig 12 shows how the averaged CS- responses vary during the course of the 80 CS- trials. For comparison, the CS+US- responses are shown as a reference. The CS- trials are shown in three blocks: trials 1-20, trials 21-60 and trials 61-80. Shown below each of the sub-panels are the corresponding averaged arousal states for each of those blocks. We would typically expect that the response to the CS- stimuli decreases as the subject learns that the CS- is never associated with the US. However, this is not the case for participant 2. There is a decrease followed by an increase in the CS- response. This decrease followed by an increase occurs both in skin conductance and the arousal state. Now the gap between the CS- and CS+US- responses is much larger in blocks 1 and 3. Therefore, when averaged, the skin conductance and state estimates are inverted. The use of the backward smoother during state estimation, and not just the forward filter, likely affects this as well. The smoother causes future estimates to affect past estimates. Consequently, the larger gap in block 3 affects the earlier estimates as well. If the method we present were implemented on a wearable device for emotion monitoring, the effect of the future on past values could be reduced by running the EM algorithm on smaller segments of data instead of on longer segments. The use of larger data segments however, is likely to make the estimate smoother.

### Selection of heart rate model parameters

We select the *θ*_*i*_ coefficients and *η* separately. While this procedure eases computation (the *θ*_*i*_’s no longer have to be repeatedly estimated at the M-step until convergence), it also creates the challenge of having to optimize both types of parameters simultaneously. To illustrate, the *θ*_*i*_ coefficients are calculated offline via maximum likelihood. This may give rise to a KS plot indicating a reasonably good fit to the heartbeat observations. However, the inclusion of *ηx*_*k*_ into the HDIG mean during state estimation alters the KS plot and the KS distance. Therefore, *η* has to be finally selected based on a trade-off of maximizing ${\bar{Q}}_{2}$ subject to the KS plot remaining within or close to the 95% confidence bounds. Moreover, the separate selection of the *θ*_*i*_’s and *η* can also give rise to numerical issues; this can especially occur at larger *η* values. As noted above, the HDIG CIF involves derivatives and integrals over small numbers. Consequently, the Newton-Raphson method used to solve Eq (15) can go into infeasible regions. The HDIG model we use here is computationally demanding. The use of a simpler probability density function to model RR-intervals may permit parameters related to heart rate to be estimated simultaneously at the M-step. This would partly eliminate issues arising due to the need to separately optimize model parameters.

Alternatively, the *θ*_*i*_ coefficients could be considered as additional states. This would permit simultaneous estimation of sympathetic arousal and the *θ*_*i*_’s. Additionally, it would also allow the *θ*_*i*_’s to be time-varying and account for some of the HRV stochasticity. In this case, the state vector to be estimated would consist of both *x*_*k*_ and the time-varying *θ*_*i*_’s. Barbieri et al. estimated a vector consisting of only the *θ*_*i*_ coefficients via Bayesian filtering in [75]. Their model could be extended to include *x*_*k*_.

### State-space model

We have used a single indicator function to model the effect of the CS+, CS- and US stimuli. In the data set used here, the CS+ and CS- were simple shapes that appeared on a computer screen. More complex stimuli have also been used in fear conditioning (e.g. complex sounds [92]). In addition, the CS+ and CS- cues can be chosen to reinforce a particular emotion (e.g. [93–95]). For instance, the CS+ stimuli could be the image of a fearful face (thus reinforcing the anticipated fear of the US) while the CS- could be a neutral face. Our current model does not distinguish between these variations in the type of stimuli as it uses a single indicator function. A further extension would be to use stimulus-specific indicator functions in the state equation, i.e.,

$$\begin{array}{cc} x_{k} & =\rho x_{k-1}+\alpha_{CS+}I_{k,CS+}+\alpha_{CS-}I_{k,CS-}++\alpha_{US}I_{k,US}+\varepsilon_{k} \end{array}$$ (33)

and determine each of the *α* coefficients separately at the M-step. This would, however, come at the expense of added computational complexity.

### Model validation and feature selection

The sympathetic arousal *x*_*k*_ that we estimate is unobserved. Consequently, we rely on a qualitative form of validation rather than a quantitative one. Here we observe the general similarity between the averaged *x*_*k*_ and skin conductance in different trial conditions as a means of validation on experimental data. Nerves from the sympathetic branch of the nervous system innervate a number of locations within the human body (e.g. skin, heart, bronchi, eye). We could therefore record signals/features from any location innervated by sympathetic nerve fibers, and treat them as the set of observations *y*_*k*_ with which to estimate *x*_*k*_ in typical state-space fashion. Thus, our choice of features here is largely based on the literature and human physiology. As we noted above, sympathetic fibers innervate the sweat glands, and the rate of SCR occurrence, the SCR amplitudes and the tonic level are the most commonly used skin conductance-related measures of sympathetic arousal [40]. Moreover, increased sympathetic drive increases heart rate. While frequency-domain features of heart rate could have been extracted, there is a lack of consensus regarding the precise interpretation of particular HRV spectral bands (the interpretation of the low frequency band, thought to reflect sympathetic activity, in particular, has been controversial [96]). There is more agreement that sympathetic drive increases heart rate (a relatively straight-forward time-domain feature).

A skin conductance signal comprises of both a slow-varying tonic component and a fast-varying phasic component. The phasic component consists of the series of SCRs which are reflections of instantaneous sympathetic nervous activity. Currently, we use both a log transformation and an interpolation over the SCR amplitudes for correcting skewness and artificially deriving a continuous-valued signal *r*_*k*_ with which to estimate *x*_*k*_. This can lead to a loss in the physiological intuition underlying the phasic SCRs—namely the *instantaneous* sympathetic activation they represent. This can especially be seen if two large SCRs separated in time occur. The interpolation will give rise to a continuous-valued *r*_*k*_ that remains high all throughout the region in-between the two SCRs and the decrease in sympathetic arousal in the middle is lost. Thus a better way to model the phasic SCR occurrences and their amplitudes would be to consider them as forming a marked point process, i.e., a point process where the events are associated with an amplitude. This is a future direction of research.

### Application to real-world scenarios

Anxiety, stress and trauma-related disorders affect a sizeable number of people and incur significant costs to both the individual patient and to society [11–13]. Disorders such as PTSD, which have a higher incidence among combat veterans [97, 98], involve a pathological condition related to a prolonged or heightened activation of the sympathetic nervous system [8, 9]. Symptoms of this elevated sympathetic arousal include irritability, an exaggerated startle response, hypervigilance and sleeping difficulties [99]. Patients diagnosed with anxiety disorders also have elevated sympathetic tones [7]. Current healthcare systems largely function in a location-centric manner, i.e., patients come to centralized locations to receive care once they are ill. Remote health monitoring reflects a gradual transition away from this model with an increased focus on the individual patient. In this model, wearable devices help keep track of a patient’s condition and provide clinical decision support, thus helping reduce healthcare costs. Our state-space algorithm, which continually tracks the level of sympathetic arousal over time, could be embedded in a wearable device and used to remotely monitor patients diagnosed with pathological fear or anxiety disorders. It could also be used to monitor patients with major depression, which is typically associated with abnormally low levels of arousal [100]. Furthermore, our approach has the advantage of being unsupervised and therefore does not require expert-labeled data for each individual patient.

Changes occur in the human body over time (e.g. due to aging, disease onset, changes in social situations). As such, models trained on data need to be updated continually. Our current state-space method functions offline due to a need to perform both forward filtering and backward smoothing, and the estimated model parameters are fixed within that particular duration of time. One possibility for adapting to the inherent stochasticity of the human body would be to re-train the models from time to time. In a real-world application, we could just run the forward filter of the E-step for providing a continual estimate of a person’s arousal level. The full EM procedure could be run in the background from time to time so that the model updates periodically. This periodic re-training would allow the model to account for intra-subject variability. Another possibility would be to combine the current Bayesian filtering approach with reinforcement learning in order to allow the model parameters to update over time. As it stands, the model is able to account for inter-subject variability since it is trained for each individual separately, but can only account for variations in time through regular re-training.

## Conclusion

Pavlovian fear conditioning has been the focus of much study over the course of the past several decades. A better understanding of the neural basis of fear conditioning and associated physiological changes has the potential to provide important insights into emotional disorders involving pathological fear and anxiety. A method to estimate the level of sympathetic arousal/activation, which plays a crucial role in the fear response, could also be beneficial in treating patients diagnosed with these disorders. We present an EM-based state-space model that utilizes skin conductance and heart rate features to do so, and evaluate it on both simulated and experimental data. Results on simulated data show the ability to accurately recover an unobserved state variable *x*_*k*_ from a binary variable, two continuous variables and a spiking-type variable. As a mathematical modeling contribution, this is an extension to [43] which estimated a cognitive learning state from one binary variable, one continuous variable and a spiking-type variable. Experimental evaluation of our model was performed on a fear conditioning experiment. Trial-averaged skin conductance values are frequently compared in fear conditioning experiments. Our algorithm’s state estimates show a general agreement with the averaged skin conductance values between different trial conditions. However, there is less separation between the CS+US- and CS- trials. This may be due to trace fear conditioning eliciting weaker responses compared to typical delay fear conditioning [17] or an insufficient US strength. Thus, the model suggests a preliminary line of evidence for estimating sympathetic arousal from binary, continuous and spiking-type observations taken from both the skin and the heart (organs which are innervated by sympathetic nerve fibers) using state-space methods. The state-space formulation presented here relates an internal brain state to observed physiological phenomena. As such it could find applications in wearable healthcare for remotely monitoring patients diagnosed with certain types of neuropsychiatric disorders or in general wellness applications such as stress management [101].

## Supporting information

S1 Appendix EM algorithm derivations and supplementary data. (PDF)

## References

[1] Hall JE. Guyton and Hall textbook of medical physiology. Elsevier Health Sciences; 2016.

[2] LeDoux JE. Emotion circuits in the brain. Annual Review of Neuroscience. 2000;23(1):155–184. 10.1146/annurev.neuro.23.1.155 10845062

[3] Rolls ET. On the brain and emotion. Behavioral and Brain Sciences. 2000;23(2):219–228. 10.1017/S0140525X00002429 11301577

[4] Bechara A, Damasio H, Damasio AR. Emotion, decision making and the orbitofrontal cortex. Cerebral Cortex. 2000;10(3):295–307. 10.1093/cercor/10.3.295 10731224

[5] García-Cabezas MÁ, Barbas H. Anterior cingulate pathways may affect emotions through orbitofrontal cortex. Cerebral Cortex. 2017;27(10):4891–4910. 10.1093/cercor/bhw284 27655930 PMC6075591

[6] Diagnostic and statistical manual of mental disorders: DSM-5. Fifth edition ed. Arlington, VA: American Psychiatric Association; 2013.

[7] Pohjavaara P, Telaranta T, Väisänen E. The role of the sympathetic nervous system in anxiety: is it possible to relieve anxiety with endoscopic sympathetic block? Nordic Journal of Psychiatry. 2003;57(1):55–60. 10.1080/08039480310000266 12745792

[8] Yehuda R, Southwick SM, Giller EL, Ma X, Mason JW. Urinary catecholamine excretion and severity of PTSD symptoms in Vietnam combat veterans. Journal of Nervous and Mental Disease. 1992;. 10.1097/00005053-199205000-00006 1583475

[9] Pervanidou P. Biology of post-traumatic stress disorder in childhood and adolescence. Journal of Neuroendocrinology. 2008;20(5):632–638. 10.1111/j.1365-2826.2008.01701.x 18363804

[10] Pitman RK, Rasmusson AM, Koenen KC, Shin LM, Orr SP, Gilbertson MW, et al Biological studies of post-traumatic stress disorder. Nature Reviews Neuroscience. 2012;13(11):769–787. 10.1038/nrn3339 23047775 PMC4951157

[11] Kessler RC, Berglund P, Demler O, Jin R, Merikangas KR, Walters EE. Lifetime prevalence and age-of-onset distributions of DSM-IV disorders in the National Comorbidity Survey Replication. Archives of General Psychiatry. 2005;62(6):593–602. 10.1001/archpsyc.62.6.593 15939837

[12] Nutt D, de Miguel BG, Davies SJ. Phenomenology of anxiety disorders. Handbook of Behavioral Neuroscience. 2008;17:365–393. 10.1016/S1569-7339(07)00017-3

[13] Leon AC, Portera L, Weissman MM. The social costs of anxiety disorders. The British Journal of Psychiatry. 1995;166(S27):19–22. 10.1192/S0007125000293355 7794589

[14] Dvir M, Horovitz O, Aderka IM, Shechner T. Fear conditioning and extinction in anxious and non-anxious youth: A meta-analysis. Behaviour Research and Therapy. 2019; p. 103431 10.1016/j.brat.2019.103431 31352065

[15] Shin LM, Liberzon I. The neurocircuitry of fear, stress, and anxiety disorders. Neuropsychopharmacology. 2010;35(1):169 10.1038/npp.2009.83 19625997 PMC3055419

[16] Maren S. Neurobiology of Pavlovian fear conditioning. Annual Review of Neuroscience. 2001;24(1):897–931. 10.1146/annurev.neuro.24.1.897 11520922

[17] Milad MR, Igoe S, Orr SP. Fear conditioning in rodents and humans In: Animal Models of Behavioral Analysis. Springer; 2011 p. 111–132.

[18] Lipp OV. Human fear learning: Contemporary procedures and measurement. Fear and Learning: From Basic Processes to Clinical Implications. 2006;(2001):37–51.

[19] VanElzakker MB, Dahlgren MK, Davis FC, Dubois S, Shin LM. From Pavlov to PTSD: the extinction of conditioned fear in rodents, humans, and anxiety disorders. Neurobiology of Learning and Memory. 2014;113:3–18. 10.1016/j.nlm.2013.11.014 24321650 PMC4156287

[20] Linnman C, Zeffiro TA, Pitman RK, Milad MR. An fMRI study of unconditioned responses in post-traumatic stress disorder. Biology of Mood & Anxiety Disorders. 2011;1(1):8 10.1186/2045-5380-1-8 22738227 PMC3384234

[21] Milad MR, Pitman RK, Ellis CB, Gold AL, Shin LM, Lasko NB, et al Neurobiological basis of failure to recall extinction memory in posttraumatic stress disorder. Biological Psychiatry. 2009;66(12):1075–1082. 10.1016/j.biopsych.2009.06.026 19748076 PMC2787650

[22] Schneider F, Weiss U, Kessler C, Müller-Gärtner HW, Posse S, Salloum JB, et al Subcortical correlates of differential classical conditioning of aversive emotional reactions in social phobia. Biological Psychiatry. 1999;45(7):863–871. 10.1016/s0006-3223(98)00269-8 10202574

[23] Benedek M, Kaernbach C. Decomposition of skin conductance data by means of nonnegative deconvolution. Psychophysiology. 2010;47(4):647–658. 10.1111/j.1469-8986.2009.00972.x 20230512 PMC2904901

[24] Amin MR, Faghih RT. Sparse deconvolution of electrodermal activity via continuous-time system identification. IEEE Transactions on Biomedical Engineering. 2019;66(9):2585–2595. 10.1109/TBME.2019.2892352 30629490

[25] Amin MR, Faghih RT. Inferring autonomic nervous system stimulation from hand and foot skin conductance measurements. In: 52nd Asilomar Conference on Signals, Systems, and Computers. IEEE; 2018. p. 655–660.

[26] Jain S, Oswal U, Xu KS, Eriksson B, Haupt J. A compressed sensing based decomposition of electrodermal activity signals. IEEE Transactions on Biomedical Engineering. 2016;64(9):2142–2151. 10.1109/TBME.2016.2632523 27893381

[27] Baczkowski BM, Johnstone T, Walter H, Erk S, Veer IM. Sliding-window analysis tracks fluctuations in amygdala functional connectivity associated with physiological arousal and vigilance during fear conditioning. NeuroImage. 2017;153:168–178. 10.1016/j.neuroimage.2017.03.022 28300639

[28] Castegnetti G, Tzovara A, Staib M, Paulus PC, Hofer N, Bach DR. Modeling fear-conditioned bradycardia in humans. Psychophysiology. 2016;53(6):930–939. 10.1111/psyp.12637 26950648 PMC4869680

[29] Gliner JA, Browe AC, Horvath SM. Hemodynamic changes as a function of classical aversive conditioning in human subjects. Psychophysiology. 1977;14(3):281–286. 10.1111/j.1469-8986.1977.tb01176.x 854557

[30] Klorman R, Ryan RM. Heart rate, contingent negative variation, and evoked potentials during anticipation of affective stimulation. Psychophysiology. 1980;17(6):513–523. 10.1111/j.1469-8986.1980.tb02290.x 7443917

[31] Furedy JJ, Poulos CX. Heart-rate decelerative Pavlovian conditioning with tilt as UCS: Towards behavioural control of cardiac dysfunction. Biological Psychology. 1976;4(2):93–105. 10.1016/0301-0511(76)90010-7 1276306

[32] Orr SP, Metzger LJ, Lasko NB, Macklin ML, Peri T, Pitman RK. De novo conditioning in trauma-exposed individuals with and without posttraumatic stress disorder. Journal of Abnormal Psychology. 2000;109(2):290 10.1037/0021-843X.109.2.290 10895567

[33] Jovanovic T, Keyes M, Fiallos A, Myers KM, Davis M, Duncan EJ. Fear potentiation and fear inhibition in a human fear-potentiated startle paradigm. Biological Psychiatry. 2005;57(12):1559–1564. 10.1016/j.biopsych.2005.02.025 15953493

[34] Norrholm SD, Jovanovic T, Vervliet B, Myers KM, Davis M, Rothbaum BO, et al Conditioned fear extinction and reinstatement in a human fear-potentiated startle paradigm. Learning & Memory. 2006;13(6):681–685. 10.1101/lm.393906 17142300 PMC3746591

[35] Orr SP, Metzger LJ, Lasko NB, Macklin ML, Hu FB, Shalev AY, et al Physiologic responses to sudden, loud tones in monozygotic twins discordant for combat exposure: association with posttraumatic stress disorder. Archives of General Psychiatry. 2003;60(3):283–288. 10.1001/archpsyc.60.3.283 12622661

[36] Sevenster D, Beckers T, Kindt M. Fear conditioning of SCR but not the startle reflex requires conscious discrimination of threat and safety. Frontiers in Behavioral Neuroscience. 2014;8:32 10.3389/fnbeh.2014.00032 24616672 PMC3937874

[37] Russell JA. A circumplex model of affect. Journal of Personality and Social Psychology. 1980;39(6):1161 10.1037/h0077714

[38] Alpers GW, Ruhleder M, Walz N, Mühlberger A, Pauli P. Binocular rivalry between emotional and neutral stimuli: A validation using fear conditioning and EEG. International Journal of Psychophysiology. 2005;57(1):25–32. 10.1016/j.ijpsycho.2005.01.008 15893834

[39] Low PA. Chapter 51—Sweating In: Robertson D, Biaggioni I, Burnstock G, Low PA, Paton JFR, editors. Primer on the Autonomic Nervous System (Third Edition). third edition ed. San Diego: Academic Press; 2012 p. 249—251.

[40] Kreibig SD. Autonomic nervous system activity in emotion: A review. Biological Psychology. 2010;84(3):394–421. 10.1016/j.biopsycho.2010.03.010 20371374

[41] Wallentin M, Nielsen AH, Vuust P, Dohn A, Roepstorff A, Lund TE. Amygdala and heart rate variability responses from listening to emotionally intense parts of a story. Neuroimage. 2011;58(3):963–973. 10.1016/j.neuroimage.2011.06.077 21749924

[42] Wickramasuriya DS, Faghih RT. A Bayesian filtering approach for tracking arousal from binary and continuous skin conductance features. IEEE Transactions on Biomedical Engineering. 2019 10.1109/TBME.2019.2945579 31603767

[43] Coleman TP, Yanike M, Suzuki WA, Brown EN. A mixed-filter algorithm for dynamically tracking learning from multiple behavioral and neurophysiological measures In: The dynamic brain: An exploration of neuronal variability and its functional significance. Oxford Univ. Press; 2011 p. 1–16.

[44] Mahan AL, Ressler KJ. Fear conditioning, synaptic plasticity and the amygdala: Implications for posttraumatic stress disorder. Trends in Neurosciences. 2012;35(1):24–35. 10.1016/j.tins.2011.06.007 21798604 PMC3206195

[45] Wickramasuriya DS, Amin MR, Faghih RT. Skin conductance as a viable alternative for closing the deep brain stimulation loop in neuropsychiatric disorders. Frontiers in Neuroscience. 2019;13:780 10.3389/fnins.2019.00780 31447627 PMC6692489

[46] Tzovara A, Hofer N, Bach DR, Castegnetti G, Gerster S, Korn CW, et al. PsPM-TC: SCR, ECG, EMG and respiration measurements in a discriminant trace fear conditioning task with visual CS and electrical US.; 2018. Available from: 10.5281/zenodo.1404810.

[47] Castegnetti G, Tzovara A, Staib M, Gerster S, Bach DR. Assessing fear learning via conditioned respiratory amplitude responses. Psychophysiology. 2017;54(2):215–223. 10.1111/psyp.12778 27933608 PMC6001548

[48] Tzovara A, Korn CW, Bach DR. Human Pavlovian fear conditioning conforms to probabilistic learning. PLoS Computational Biology. 2018;14(8):e1006243 10.1371/journal.pcbi.1006243 30169519 PMC6118355

[49] Sano A, Phillips AJ, Amy ZY, McHill AW, Taylor S, Jaques N, et al. Recognizing academic performance, sleep quality, stress level, and mental health using personality traits, wearable sensors and mobile phones. In: IEEE 12th International Conference on Wearable and Implantable Body Sensor Networks (BSN). IEEE; 2015. p. 1–6. 10.1109/BSN.2015.7299420 PMC5431072 28516162

[50] Greco A, Valenza G, Lanata A, Scilingo EP, Citi L. cvxEDA: A convex optimization approach to electrodermal activity processing. IEEE Transactions on Biomedical Engineering. 2015;63(4):797–804. 10.1109/TBME.2015.2474131 26336110

[51] Smith AC, Frank LM, Wirth S, Yanike M, Hu D, Kubota Y, et al Dynamic analysis of learning in behavioral experiments. Journal of Neuroscience. 2004;24(2):447–461. 10.1523/JNEUROSCI.2908-03.2004 14724243 PMC6729979

[52] Prerau MJ, Smith AC, Eden UT, Kubota Y, Yanike M, Suzuki W, et al Characterizing learning by simultaneous analysis of continuous and binary measures of performance. Journal of Neurophysiology. 2009;102(5):3060–3072. 10.1152/jn.91251.2008 19692505 PMC2777819

[53] Deng X, Faghih RT, Barbieri R, Paulk AC, Asaad WF, Brown EN, et al. Estimating a dynamic state to relate neural spiking activity to behavioral signals during cognitive tasks. In: 37th Annual International Conference of the IEEE Engineering in Medicine and Biology Society (EMBC). IEEE; 2015. p. 7808–7813. 10.1109/EMBC.2015.7320203 PMC6118213 26738103

[54] Prerau MJ, Hartnack KE, Obregon-Henao G, Sampson A, Merlino M, Gannon K, et al Tracking the sleep onset process: An empirical model of behavioral and physiological dynamics. PLoS Computational Biology. 2014;10(10):e1003866 10.1371/journal.pcbi.1003866 25275376 PMC4183428

[55] Smith AC, Brown EN. Estimating a state-space model from point process observations. Neural Computation. 2003;15(5):965–991. 10.1162/089976603765202622 12803953

[56] Critchley HD. Electrodermal responses: What happens in the brain. The Neuroscientist. 2002;8(2):132–142. 10.1177/107385840200800209 11954558

[57] Aikins DE, Johnson DC, Borelli JL, Klemanski DH, Morrissey PM, Benham TL, et al Thought suppression failures in combat PTSD: A cognitive load hypothesis. Behaviour Research and Therapy. 2009;47(9):744–751. 10.1016/j.brat.2009.06.006 19586619

[58] Laberg JC, Ellertsen B. Psychophysiological indicators of craving in alcoholics: Effects of cue exposure. British Journal of Addiction. 1987;82(12):1341–1348. 10.1111/j.1360-0443.1987.tb00437.x 3480748

[59] Kallinen K, Ravaja N. Emotion-related effects of speech rate and rising vs. falling background music melody during audio news: The moderating influence of personality. Personality and Individual Differences. 2004;37(2):275–288. 10.1016/j.paid.2003.09.002

[60] Lithari C, Frantzidis C, Papadelis C, Vivas AB, Klados M, Kourtidou-Papadeli C, et al Are females more responsive to emotional stimuli? A neurophysiological study across arousal and valence dimensions. Brain Topography. 2010;23(1):27–40. 10.1007/s10548-009-0130-5 20043199 PMC2816804

[61] Mella N, Conty L, Pouthas V. The role of physiological arousal in time perception: psychophysiological evidence from an emotion regulation paradigm. Brain and Cognition. 2011;75(2):182–187. 10.1016/j.bandc.2010.11.012 21145643

[62] Nagai Y, Critchley HD, Featherstone E, Trimble MR, Dolan RJ. Activity in ventromedial prefrontal cortex covaries with sympathetic skin conductance level: A physiological account of a “default mode” of brain function. Neuroimage. 2004;22(1):243–251. 10.1016/j.neuroimage.2004.01.019 15110014

[63] Gatzke-Kopp LM, Raine A, Loeber R, Stouthamer-Loeber M, Steinhauer SR. Serious delinquent behavior, sensation seeking, and electrodermal arousal. Journal of Abnormal Child Psychology. 2002;30(5):477–486. 10.1023/a:1019816930615 12403151

[64] Barry RJ, Sokolov EN. Habituation of phasic and tonic components of the orienting reflex. International Journal of Psychophysiology. 1993;15(1):39–42. 10.1016/0167-8760(93)90093-5 8407432

[65] McCullagh P, Nelder JA. Generalized linear models. vol. 37 CRC press; 1989.

[66] Braithwaite JJ, Watson DG, Jones R, Rowe M. A guide for analysing electrodermal activity (EDA) & skin conductance responses (SCRs) for psychological experiments. Psychophysiology. 2013;49(1):1017–1034.

[67] Boucsein W. Electrodermal activity. Springer Science & Business Media; 2012.

[68] Bach DR, Flandin G, Friston KJ, Dolan RJ. Modelling event-related skin conductance responses. International Journal of Psychophysiology. 2010;75(3):349–356. 10.1016/j.ijpsycho.2010.01.005 20093150 PMC2877881

[69] Dawson ME, Schell AM, Filion DL. The electrodermal system. Handbook of Psychophysiology. 2007;2:200–223.

[70] Drew RC, Sinoway LI. Autonomic control of the heart In: Primer on the autonomic nervous system. Elsevier; 2012 p. 177–180.

[71] Berntson GG, Cacioppo JT, Quigley KS. The metrics of cardiac chronotropism: Biometric perspectives. Psychophysiology. 1995;32(2):162–171. 10.1111/j.1469-8986.1995.tb03308.x 7630981

[72] Berntson GG, Thomas Bigger J Jr, Eckberg DL, Grossman P, Kaufmann PG, Malik M, et al Heart rate variability: origins, methods, and interpretive caveats. Psychophysiology. 1997;34(6):623–648. 10.1111/j.1469-8986.1997.tb02140.x 9401419

[73] Barbieri R, Matten EC, Alabi AA, Brown EN. A point-process model of human heartbeat intervals: new definitions of heart rate and heart rate variability. American Journal of Physiology-Heart and Circulatory Physiology. 2005;288(1):H424–H435. 10.1152/ajpheart.00482.2003 15374824

[74] Stanley GB, Poolla K, Siegel RA. Threshold modeling of autonomic control of heart rate variability. IEEE Transactions on Biomedical Engineering. 2000;47(9):1147–1153. 10.1109/10.867918 11008415

[75] Barbieri R, Brown EN. Analysis of heartbeat dynamics by point process adaptive filtering. IEEE Transactions on Biomedical Engineering. 2006;53(1):4–12. 10.1109/tbme.2005.859779 16402597

[76] Boardman A, Schlindwein FS, Rocha AP. A study on the optimum order of autoregressive models for heart rate variability. Physiological Measurement. 2002;23(2):325 10.1088/0967-3334/23/2/308 12051304

[77] Pichon A, Roulaud M, Antoine-Jonville S, de Bisschop C, Denjean A. Spectral analysis of heart rate variability: Interchangeability between autoregressive analysis and fast Fourier transform. Journal of Electrocardiology. 2006;39(1):31–37. 10.1016/j.jelectrocard.2005.08.001 16387047

[78] Barbieri R, Brown EN. Application of dynamic point process models to cardiovascular control. Biosystems. 2008;93(1-2):120–125. 10.1016/j.biosystems.2008.03.011 18515000 PMC2561955

[79] Eden UT, Srinivasan L, Sarma SV. Nueral signal processing tutorial II: Point process model estimation and goodness-of-fit analysis In: Mitra P, editor. Neural Signal Processing: Quantitative Analysis of Neural Activity. Washington DC: Society for Neuroscience; 2008 p. 79–87.

[80] Mendel JM. Lessons in estimation theory for signal processing, communications and control. Pearson Education; 1995.

[81] Jong PD, Mackinnon MJ. Covariances for smoothed estimates in state space models. Biometrika. 1988;75(3):601–602. 10.1093/biomet/75.3.601

[82] Wickramasuriya DS, Qi C, Faghih RT. A state-space approach for detecting stress from electrodermal activity. In: 40th Annual International Conference of the IEEE Engineering in Medicine and Biology Society (EMBC); 2018. p. 3562–3567. 10.1109/EMBC.2018.8512928 30441148

[83] Wickramasuriya DS, Faghih RT. A novel filter for tracking real-world cognitive stress using multi-time-scale point process observations. In: 41st Annual International Conference of the IEEE Engineering in Medicine and Biology Society (EMBC); 2019. p. 599–602. 10.1109/EMBC.2019.8857917 31945969

[84] Brown EN, Barbieri R, Ventura V, Kass RE, Frank LM. The time-rescaling theorem and its application to neural spike train data analysis. Neural Computation. 2002;14(2):325–346. 10.1162/08997660252741149 11802915

[85] Eden UT, Frank LM, Barbieri R, Solo V, Brown EN. Dynamic analysis of neural encoding by point process adaptive filtering. Neural Computation. 2004;16(5):971–998. 10.1162/089976604773135069 15070506

[86] Barbieri R, Quirk MC, Frank LM, Wilson MA, Brown EN. Construction and analysis of non-Poisson stimulus-response models of neural spiking activity. Journal of Neuroscience Methods. 2001;105(1):25–37. 10.1016/s0165-0270(00)00344-7 11166363

[87] Koyama S, Kass RE. Spike train probability models for stimulus-driven leaky integrate-and-fire neurons. Neural Computation. 2008;20(7):1776–1795. 10.1162/neco.2008.06-07-540 18336078 PMC2715549

[88] Chen Z, Brown EN, Barbieri R. Assessment of autonomic control and respiratory sinus arrhythmia using point process models of human heart beat dynamics. IEEE Transactions on Biomedical Engineering. 2009;56(7):1791–1802. 10.1109/TBME.2009.2016349 19272971 PMC2804879

[89] Prechelt L. Early stopping-but when? In: Neural networks: Tricks of the trade. Springer; 1998 p. 55–69.

[90] Malem-Shinitski N, Zhang Y, Gray DT, Burke SN, Smith AC, Barnes CA, et al A separable two-dimensional random field model of binary response data from multi-day behavioral experiments. Journal of Neuroscience Methods. 2018;307:175–187. 10.1016/j.jneumeth.2018.04.006 29679704 PMC6417888

[91] Yousefi A, Gillespie AK, Guidera JA, Karlsson M, Frank LM, Eden UT. Efficient decoding of multi-dimensional signals from population spiking activity using a Gaussian mixture particle filter. IEEE Transactions on Biomedical Engineering. 2019;66(12):3486–3498. 10.1109/TBME.2019.2906640 30932819 PMC7516928

[92] Staib M, Bach DR. Stimulus-invariant auditory cortex threat encoding during fear conditioning with simple and complex sounds. NeuroImage. 2018;166:276–284. 10.1016/j.neuroimage.2017.11.009 29122722 PMC5770332

[93] Regan M, Howard R. Fear conditioning, preparedness, and the contingent negative variation. Psychophysiology. 1995;32(3):208–214. 10.1111/j.1469-8986.1995.tb02950.x 7784529

[94] Dunsmoor JE, Mitroff SR, LaBar KS. Generalization of conditioned fear along a dimension of increasing fear intensity. Learning & Memory. 2009;16(7):460–469. 10.1101/lm.1431609 19553384 PMC2704105

[95] Mueller EM, Sperl MF, Panitz C. Aversive imagery causes De Novo fear conditioning. Psychological Science. 2019; p. 0956797619842261. 10.1177/0956797619842261 PMC6651612 31150589

[96] Rahman F, Pechnik S, Gross D, Sewell L, Goldstein DS. Low frequency power of heart rate variability reflects baroreflex function, not cardiac sympathetic innervation. Clinical Autonomic Research. 2011;21(3):133–141. 10.1007/s10286-010-0098-y 21279414 PMC3094491

[97] Kang HK, Natelson BH, Mahan CM, Lee KY, Murphy FM. Post-traumatic stress disorder and chronic fatigue syndrome-like illness among Gulf War veterans: a population-based survey of 30,000 veterans. American Journal of Epidemiology. 2003;157(2):141–148. 10.1093/aje/kwf187 12522021

[98] Tanielian TL, Tanielian T, Jaycox L. Invisible wounds of war: Psychological and cognitive injuries, their consequences, and services to assist recovery. vol. 1 Rand Corporation; 2008.

[99] Yehuda R, LeDoux J. Response variation following trauma: a translational neuroscience approach to understanding PTSD. Neuron. 2007;56(1):19–32. 10.1016/j.neuron.2007.09.006 17920012

[100] Moratti S, Rubio G, Campo P, Keil A, Ortiz T. Hypofunction of right temporoparietal cortex during emotional arousal in depression. Archives of General Psychiatry. 2008;65(5):532–541. 10.1001/archpsyc.65.5.532 18458205

[101] Azgomi HF, Wickramasuriya DS, Faghih RT. State-space modeling and Fuzzy feedback control of cognitive stress. In: 41st Annual International Conference of the IEEE Engineering in Medicine and Biology Society (EMBC); 2019. p. 6327–6330. 10.1109/EMBC.2019.8857904 31947289

