# Supplementary material for: A mixed filter algorithm for sympathetic arousal tracking from skin conductance and heart rate measurements in Pavlovian fear conditioning
Source: PLoS One. 2020 Apr 23;15(4):e0231659. doi: 10.1371/journal.pone.0231659 (PMC7179889; doi:10.1371/journal.pone.0231659)
Supplement: S1 Appendix — (PDF) [file pone.0231659.s001.pdf]

EM algorithm derivations and supplementary data

1 Algorithm derivations

1.1 Derivation of E-step filter updates

1.1.1 Probability mass function of m_k

$$\begin{aligned}
P(m_k|x_k) &= p_k^{m_k}(1-p_k)^{1-m_k} \\
&= \exp\Big\{\log\Big[p_k^{m_k}(1-p_k)^{1-m_k}\Big]\Big\} \\
&= \exp\Big[m_k \log(p_k) + (1-m_k)\log(1-p_k)\Big] \\
&= \exp\left[m_k \log\left(\frac{p_k}{1-p_k}\right) + \log(1-p_k)\right] \\
&= \exp\left[m_k(\beta_0 + \beta_1 x_k) + \log\left(\frac{1}{1+e^{\beta_0+\beta_1 x_k}}\right)\right] \qquad (1)
\end{aligned}$$

1.1.2 Mean and variance of the posterior density function $p(x_k|y^k)$

We follow an approach similar to [1] in deriving the filter updates. Recall that linear models are assumed to relate sympathetic arousal to the phasic-derived and tonic components.

$$r_k = \gamma_0 + \gamma_1 x_k + v_k \qquad (2)$$

$$s_k = \delta_0 + \delta_1 x_k + w_k \qquad (3)$$

We take the two noise terms v_k and w_k to be independent of each other. Consequently, the density functions $p(r_k|x_k)$ and $p(s_k|x_k)$ conditioned on already having observed x_k are independent of each other in the following derivation.

$$\begin{aligned}
p(x_k|y^k) &= \frac{p(x_k|y^{k-1})p(y_k|x_k)}{p(y_k|y^{k-1})} \\
&= \frac{p(x_k|y^{k-1})P(m_k|x_k)p(r_k|x_k)p(s_k|x_k)P(n_{k,1:J}|x_k)}{p(y_k|y^{k-1})} \\
&\propto \exp\Bigg[\frac{-(x_k - x_{k|k-1})^2}{2\sigma^2_{k|k-1}} + m_k \log\left(\frac{p_k}{1-p_k}\right) + \log(1-p_k) \\
&\quad - \frac{(r_k - \gamma_0 - \gamma_1 x_k)^2}{2\sigma_v^2} - \frac{(s_k - \delta_0 - \delta_1 x_k)^2}{2\sigma_w^2} + \sum_{j=1}^{J} \log(\lambda_{k,j}\Delta)n_{k,j} - \lambda_{k,j}\Delta\Bigg] \qquad (4)
\end{aligned}$$

$$\begin{aligned}
\log\Big[p(x_k|y^k)\Big] &= \Bigg[\frac{-(x_k - x_{k|k-1})^2}{2\sigma^2_{k|k-1}} + m_k \log\left(\frac{p_k}{1-p_k}\right) + \log(1-p_k) \\
&\quad - \frac{(r_k - \gamma_0 - \gamma_1 x_k)^2}{2\sigma_v^2} - \frac{(s_k - \delta_0 - \delta_1 x_k)^2}{2\sigma_w^2} + \sum_{j=1}^{J} \log(\lambda_{k,j}\Delta)n_{k,j} - \lambda_{k,j}\Delta\Bigg] + \text{const} \qquad (5)
\end{aligned}$$

We take the partial derivative of the logarithm term above and set it to 0 to solve for the mean.

$$\frac{\partial}{\partial x_k} \log \Big[p(x_k|y^k)\Big] = \frac{-(x_k - x_{k|k-1})}{\sigma^2_{k|k-1}} + \beta_1(m_k - p_k) + \frac{\gamma_1(r_k - \gamma_0 - \gamma_1 x_k)}{\sigma^2_v} + \frac{\delta_1(s_k - \delta_0 - \delta_1 x_k)}{\sigma^2_w} + \sum_{j=1}^{J} \frac{1}{\lambda_{k,j|k}} \frac{\partial \lambda_{k,j|k}}{\partial x_k}(n_{k,j} - \lambda_{k,j|k}\Delta) = 0. \tag{6}$$

Solving for x_k in the equation above provides the filter update for $x_{k|k}$. We have taken,

$$\frac{\partial p_k}{\partial x_k} = \beta_1 p_k(1 - p_k) \tag{7}$$

when calculating the partial derivative. Similarly, the second partial derivative is,

$$\begin{aligned} \frac{\partial^2}{\partial x_k^2} \log \Big[p(x_k|y^k)\Big] &= \frac{-1}{\sigma^2_{k|k-1}} - \beta_1 \frac{\partial p_k}{\partial x_k} - \frac{\gamma_1^2}{\sigma^2_v} - \frac{\delta_1^2}{\sigma^2_w} + \frac{\partial}{\partial x_k}\left[\sum_{j=1}^{J} \frac{1}{\lambda_{k,j|k}} \frac{\partial \lambda_{k,j|k}}{\partial x_k}(n_{k,j} - \lambda_{k,j|k}\Delta)\right] \\ &= \frac{-1}{\sigma^2_{k|k-1}} - \beta_1^2 p_k(1 - p_k) - \frac{\gamma_1^2}{\sigma^2_v} - \frac{\delta_1^2}{\sigma^2_w} \\ &+ \sum_{j=1}^{J}\left[\frac{1}{\lambda_{k,j|k}} \frac{\partial^2 \lambda_{k,j|k}}{\partial x_k^2}(n_{k,j} - \lambda_{k,j|k}\Delta) - \frac{n_{k,j}}{\lambda^2_{k,j|k}}\left(\frac{\partial \lambda_{k,j|k}}{\partial x_k}\right)^2\right]. \end{aligned} \tag{8}$$

The filter update for $\sigma^2_{k|k}$ is given by [1],

$$\sigma^2_{k|k} = \left\{ -\frac{\partial^2}{\partial x_k^2} \log \Big[p(x_k|y^k)\Big]\right\}^{-1}. \tag{9}$$

1.2 Derivation of the M-step updates

1.2.1 Complete data log-likelihood

Taking $\mathcal{X}^K = \{x_1, x_2, \ldots, x_K\}$, the complete data likelihood conditioned on the model parameters Θ is given by,

$$\begin{aligned} p\big(\mathcal{Y}^K, \mathcal{X}^K|\Theta\big) = \prod_{k=1}^{K} p_k^{m_k}(1 - p_k)^{1-m_k} \times \prod_{k=1}^{K} \frac{1}{\sqrt{2\pi\sigma_v^2}} e^{-\frac{(r_k - \gamma_0 - \gamma_1 x_k)^2}{2\sigma_v^2}} \\ \times \prod_{k=1}^{K} \frac{1}{\sqrt{2\pi\sigma_w^2}} e^{-\frac{(s_k - \delta_0 - \delta_1 x_k)^2}{2\sigma_w^2}} \times \prod_{k=1}^{K} e^{\sum_{j=1}^{J} \log(\lambda_{k,j}\Delta) n_{k,j} - \lambda_{k,j}\Delta} \\ \times \prod_{k=1}^{K} \frac{1}{\sqrt{2\pi\sigma_\varepsilon^2}} e^{-\frac{(x_k - \rho x_{k-1} - \alpha I_k)^2}{2\sigma_\varepsilon^2}}. \end{aligned} \tag{10}$$

The expected log-likelihood is,

$$Q = \sum_{k=1}^{K} \mathbb{E}\Big[m_k(\beta_0 + \beta_1 x_k) - \log\big(1 + e^{\beta_0 + \beta_1 x_k}\big)\Big] + \frac{(-K)}{2} \log\big(2\pi\sigma_v^2\big)$$

$$
-\sum_{k=1}^{K} \frac{\mathbb{E}\Big[(r_k-\gamma_0-\gamma_1 x_k)^2\Big]}{2\sigma_v^2} + \frac{(-K)}{2}\log\left(2\pi\sigma_w^2\right) - \sum_{k=1}^{K} \frac{\mathbb{E}\Big[(s_k-\delta_0-\delta_1 x_k)^2\Big]}{2\sigma_w^2}
$$

$$
+\sum_{k=1}^{K}\sum_{j=1}^{J}\mathbb{E}\Big[\log(\lambda_{k,j}\Delta)n_{k,j}-\lambda_{k,j}\Delta\Big] + \frac{(-K)}{2}\log\left(2\pi\sigma_\varepsilon^2\right) - \sum_{k=1}^{K} \frac{\mathbb{E}\Big[(x_k-\rho x_{k-1}-\alpha I_k)^2\Big]}{2\sigma_\varepsilon^2}. \tag{11}
$$

Following [2], we take

$$
x_{k|K} = \mathbb{E}\Big[x_k|\mathcal{Y}^K,\Theta\Big] \tag{12}
$$

$$
U_k = \mathbb{E}\Big[x_k^2|\mathcal{Y}^K,\Theta\Big] \tag{13}
$$

$$
U_{k,k+1} = \mathbb{E}\Big[x_k x_{k+1}|\mathcal{Y}^K,\Theta\Big]. \tag{14}
$$

1.2.2 M-step updates for α and ρ

Let Q_1 denote the term in Q that contains α and ρ.

$$
Q_1 = \frac{1}{2\sigma_\varepsilon^2}\sum_{k=1}^{K}\mathbb{E}\Big[(x_k-\rho x_{k-1}-\alpha I_k)^2\Big] \tag{15}
$$

While it is possible to determine the starting state x_0 as a separate parameter, we follow one of the options in [2, 3] and set $x_0 = x_1$. This permits some bias at the beginning. Therefore,

$$
Q_1 = \frac{1}{2\sigma_\varepsilon^2}\left\{\sum_{k=2}^{K}\mathbb{E}\Big[(x_k-\rho x_{k-1}-\alpha I_k)^2\Big] + \mathbb{E}\Big[(\alpha I_1)^2\Big]\right\}. \tag{16}
$$

We take the partial derivatives of Q_1 with respect to α and ρ and set them to 0 to obtain the M-step updates.

$$
\begin{aligned}
\frac{\partial Q_1}{\partial \alpha} &= \sum_{k=2}^{K}\mathbb{E}\big[-2I_k(x_k-\rho x_{k-1}-\alpha I_k)\big] + 2\alpha I_1^2 \\
0 &= -\sum_{k=2}^{K} I_k\mathbb{E}\big[x_k\big] + \rho\sum_{k=2}^{K} I_k\mathbb{E}\big[x_{k-1}\big] + \alpha\sum_{k=1}^{K} I_k^2 \\
&= -\sum_{k=2}^{K} I_k x_{k|K} + \rho\sum_{k=2}^{K} I_k x_{k-1|K} + \alpha\sum_{k=1}^{K} I_k^2 \qquad (17) \\
\frac{\partial Q_1}{\partial \rho} &= \sum_{k=2}^{K}\mathbb{E}\big[-2x_{k-1}(x_k-\rho x_{k-1}-\alpha I_k)\big] \\
0 &= -\sum_{k=2}^{K}\mathbb{E}\big[x_k x_{k-1}\big] + \rho\sum_{k=2}^{K}\mathbb{E}\big[x_{k-1}^2\big] + \alpha\sum_{k=2}^{K} I_k\mathbb{E}\big[x_{k-1}\big] \\
&= -\sum_{k=1}^{K-1} U_{k,k+1} + \rho\sum_{k=1}^{K-1} U_k + \alpha\sum_{k=2}^{K} I_k x_{k-1|K} \qquad (18) \\
& \qquad (19)
\end{aligned}
$$

The solutions to these simultaneous equations provide α and ρ.

1.2.3 M-step updates for γ_0, γ_1, δ_0 and δ_1

Let Q_2 denote the term in Q containing γ_0 and γ_1.

$$Q_2 = \sum_{k=1}^{K} \frac{\mathbb{E}\Big[(r_k - \gamma_0 - \gamma_1 x_k)^2\Big]}{2\sigma_v^2} \tag{20}$$

Taking the partial derivatives with respect to γ_0 and γ_1 yields,

$$\begin{aligned}
\frac{\partial Q_2}{\partial \gamma_0} &= \sum_{k=1}^{K} -2\mathbb{E}\big[r_k - \gamma_0 - \gamma_1 x_k\big] \\
0 &= -\sum_{k=1}^{K} r_k + \gamma_0 K + \gamma_1 \sum_{k=1}^{K} \mathbb{E}\big[x_k\big] \\
&= -\sum_{k=1}^{K} r_k + \gamma_0 K + \gamma_1 \sum_{k=1}^{K} x_{k|K} \qquad (21) \\
\frac{\partial Q_2}{\partial \gamma_1} &= \sum_{k=1}^{K} -2\mathbb{E}\big[x_k(r_k - \gamma_0 - \gamma_1 x_k)\big] \\
0 &= -\sum_{k=1}^{K} r_k \mathbb{E}\big[x_k\big] + \gamma_0 \sum_{k=1}^{K} \mathbb{E}\big[x_k\big] + \gamma_1 \sum_{k=1}^{K} \mathbb{E}\big[x_k^2\big] \\
&= -\sum_{k=1}^{K} r_k x_{k|K} + \gamma_0 \sum_{k=1}^{K} x_{k|K} + \gamma_1 \sum_{k=1}^{K} U_k. \qquad (22)
\end{aligned}$$

The solutions to these simultaneous equations provide γ_0 and γ_1. δ_0 and δ_1 may be obtained similarly from the term in Q containing s_k.

1.2.4 M-step updates for σ_v^2 and σ_w^2

Let Q_3 denote the term in Q containing σ_v^2.

$$Q_3 = \frac{-K}{2} \log\left(2\pi\sigma_v^2\right) - \sum_{k=1}^{K} \frac{\mathbb{E}\Big[(r_k - \gamma_0 - \gamma_1 x_k)^2\Big]}{2\sigma_v^2} \tag{23}$$

We take the partial derivative with respect to σ_v^2 and set it to 0.

$$\frac{\partial Q_3}{\partial \sigma_v^2} = \frac{-K}{2\sigma_v^2} + \frac{1}{2\sigma_v^4} \sum_{k=1}^{K} \mathbb{E}\Big[(r_k - \gamma_0 - \gamma_1 x_k)^2\Big] = 0 \tag{24}$$

$$\begin{aligned}
\sigma_v^2 &= \frac{1}{K} \sum_{k=1}^{K} \mathbb{E}\Big[(r_k - \gamma_0 - \gamma_1 x_k)^2\Big] \\
&= \frac{1}{K} \left\{ \sum_{k=1}^{K} r_k^2 + K\gamma_0^2 + \gamma_1^2 \sum_{k=1}^{K} \mathbb{E}\big[x_k^2\big] - 2\gamma_0 \sum_{k=1}^{K} r_k - 2\gamma_1 \sum_{k=1}^{K} r_k \mathbb{E}\big[x_k\big] + 2\gamma_0\gamma_1 \sum_{k=1}^{K} \mathbb{E}\big[x_k\big] \right\} \\
&= \frac{1}{K} \left\{ \sum_{k=1}^{K} r_k^2 + K\gamma_0^2 + \gamma_1^2 \sum_{k=1}^{K} U_k - 2\gamma_0 \sum_{k=1}^{K} r_k - 2\gamma_1 \sum_{k=1}^{K} r_k x_{k|K} + 2\gamma_0\gamma_1 \sum_{k=1}^{K} x_{k|K} \right\}. \qquad (25)
\end{aligned}$$

The update for σ_w^2 may be obtained likewise.

1.2.5 M-step update for σ_ε^2

Let Q_4 denote the term in Q containing σ_ε^2.

$$
\begin{aligned}
Q_4 &= \frac{-K}{2}\log\left(2\pi\sigma_\varepsilon^2\right) - \sum_{k=1}^{K}\frac{\mathbb{E}\Big[(x_k-\rho x_{k-1}-\alpha I_k)^2\Big]}{2\sigma_\varepsilon^2} \\
&= \frac{-K}{2}\log\left(2\pi\sigma_\varepsilon^2\right) - \sum_{k=2}^{K}\frac{\mathbb{E}\Big[(x_k-\rho x_{k-1}-\alpha I_k)^2\Big]}{2\sigma_\varepsilon^2} - \frac{\mathbb{E}\Big[(\alpha I_1)^2\Big]}{2\sigma_\varepsilon^2} \qquad (26)
\end{aligned}
$$

We take the partial derivative with respect to σ_ε^2 and set it to 0.

$$
\frac{\partial Q_4}{\partial \sigma_\varepsilon^2} = \frac{-K}{2\sigma_\varepsilon^2} + \frac{1}{2\sigma_\varepsilon^4}\sum_{k=2}^{K}\mathbb{E}\Big[(x_k-\rho x_{k-1}-\alpha I_k)^2\Big] + \frac{(\alpha I)^2}{2\sigma_\varepsilon^4} = 0 \qquad (27)
$$

$$
\begin{aligned}
\sigma_\varepsilon^2 &= \frac{1}{K}\sum_{k=2}^{K}\Big\{\mathbb{E}\big[x_k^2\big] - 2\rho\mathbb{E}\big[x_k x_{k-1}\big] + \rho^2\mathbb{E}\big[x_{k-1}^2\big] - 2\alpha I_k\mathbb{E}\big[x_k\big] + 2\alpha\rho I_k\mathbb{E}\big[x_{k-1}\big]\Big\} + \frac{\alpha^2}{K}\sum_{k=1}^{K} I_k^2 \\
&= \frac{1}{K}\left\{\sum_{k=2}^{K}U_k - 2\rho\sum_{k=1}^{K-1}U_{k,k+1} + \rho^2\sum_{k=1}^{K-1}U_k - 2\alpha\sum_{k=2}^{K}I_k x_{k|K} + 2\alpha\rho\sum_{k=2}^{K}I_k x_{k-1|K} + \alpha^2\sum_{k=1}^{K}I_k^2\right\} \qquad (28)
\end{aligned}
$$

1.2.6 M-step updates for β_0 and β_1

Let Q_5 denote the expectation term containing β_0 and β_1.

$$
Q_5 = \sum_{k=1}^{K}\mathbb{E}\Big[m_k(\beta_0+\beta_1 x_k) - \log\left(1+e^{\beta_0+\beta_1 x_k}\right)\Big] \qquad (29)
$$

We perform a Taylor expansion of the logarithm term around $x_{k|K}[1]$.

$$
\log\left(1+e^{\beta_0+\beta_1 x_k}\right) \approx \log\left(1+e^{\beta_0+\beta_1 x_{k|K}}\right) + \beta_1 p_{k|K}(x_k - x_{k|K}) + \frac{\beta_1^2}{2}p_{k|K}(1-p_{k|K})(x_k-x_{k|K})^2 \qquad (30)
$$

Taking the expected value on both sides,

$$
\begin{aligned}
\mathbb{E}\Big[\log\left(1+e^{\beta_0+\beta_1 x_k}\right)\Big] &\approx \log\left(1+e^{\beta_0+\beta_1 x_{k|K}}\right) + \beta_1 p_{k|K}\mathbb{E}\big[x_k - x_{k|K}\big] + \frac{\beta_1^2}{2}p_{k|K}(1-p_{k|K})\mathbb{E}\big[(x_k-x_{k|K})^2\big] \\
&= \log\left(1+e^{\beta_0+\beta_1 x_{k|K}}\right) + 0 + \frac{\beta_1^2}{2}p_{k|K}(1-p_{k|K})\sigma_{k|K}^2. \qquad (31)
\end{aligned}
$$

Therefore,

$$
Q_5 \approx \sum_{k=1}^{K}\left[m_k(\beta_0+\beta_1 x_{k|K}) - \log\left(1+e^{\beta_0+\beta_1 x_{k|K}}\right) - \frac{\beta_1^2}{2}p_{k|K}(1-p_{k|K})\sigma_{k|K}^2\right]. \qquad (32)
$$

Now,

$$
\frac{\partial p_{k|K}}{\partial \beta_0} = \frac{\partial}{\partial \beta_0}\left[\frac{1}{1+e^{-(\beta_0+\beta_1 x_{k|K})}}\right]
$$

$$= \frac{(-1)}{\Big[1+e^{-(\beta_0+\beta_1 x_{k|K})}\Big]^2} \times \Big[-e^{-(\beta_0+\beta_1 x_{k|K})}\Big]$$
$$= p_{k|K}(1-p_{k|K}). \tag{33}$$

And similarly,

$$\frac{\partial p_{k|K}}{\partial \beta_1} = p_{k|K}(1-p_{k|K})x_{k|K}. \tag{34}$$

Taking the partial derivative w.r.t. β_0,

$$\frac{\partial Q_5}{\partial \beta_0} = \sum_{k=1}^{K} \left\{ m_k - p_{k|K} - \frac{\beta_1^2 \sigma_{k|K}^2}{2} \frac{\partial}{\partial \beta_0}\Big[p_{k|K}(1-p_{k|K})\Big] \right\}$$
$$0 = \sum_{k=1}^{K} \left[m_k - p_{k|K} - \frac{\beta_1^2 \sigma_{k|K}^2}{2}(1-p_{k|K})(1-2p_{k|K})p_{k|K} \right]. \tag{35}$$

And similarly for β_1 we arrive at,

$$\frac{\partial Q_5}{\partial \beta_1} = \sum_{k=1}^{K} \Bigg[m_k x_{k|K} - x_{k|K} p_{k|K}$$
$$- \frac{\beta_1 \sigma_{k|K}^2}{2} p_{k|K}(1-p_{k|K})\big[2+\beta_1 x_{k|K}(1-2p_{k|K})\big] \Bigg]$$
$$= 0 \tag{36}$$

1.2.7 Approximation for the expectation term containing $\lambda_{k,j}$

Let Q_6 denote the expectation term containing $\lambda_{k,j}$.

$$Q_6 = \sum_{k=1}^{K} \sum_{j=1}^{J} \mathbb{E}\Big[\log(\lambda_{k,j}\Delta)n_{k,j} - \lambda_{k,j}\Delta\Big] \tag{37}$$

We perform a Taylor expansion of the summed term around $x_{k|K}$ [1].

$$\log(\lambda_{k,j}\Delta)n_{k,j} - \lambda_{k,j}\Delta \approx \log(\lambda_{k,j|K}\Delta)n_{k,j} - \lambda_{k,j|K}\Delta + \frac{1}{\lambda_{k,j|K}} \frac{\partial \lambda_{k,j|K}}{\partial x_k}(n_{k,j} - \lambda_{k,j|K}\Delta)(x_k - x_{k|K})$$
$$+ \frac{1}{2}\left[\frac{1}{\lambda_{k,j|K}} \frac{\partial^2 \lambda_{k,j|K}}{\partial x_k^2}(n_{k,j} - \lambda_{k,j|K}\Delta) - \frac{n_{k,j}}{\lambda_{k,j|K}^2}\left(\frac{\partial \lambda_{k,j|K}}{\partial x_k}\right)^2 \right](x_k - x_{k|K})^2 \tag{38}$$

Taking the expected value on both sides,

$$\mathbb{E}\big[\log(\lambda_{k,j}\Delta)n_{k,j} - \lambda_{k,j|K}\Delta\big] \approx \log(\lambda_{k,j|K}\Delta)n_{k,j} - \lambda_{k,j|K}\Delta + \frac{1}{\lambda_{k,j|K}} \frac{\partial \lambda_{k,j|K}}{\partial x_k}(n_{k,j} - \lambda_{k,j|K}\Delta)\mathbb{E}\big[x_k - x_{k|K}\big]$$
$$+ \frac{1}{2}\left[\frac{1}{\lambda_{k,j|K}} \frac{\partial^2 \lambda_{k,j|K}}{\partial x_k^2}(n_{k,j} - \lambda_{k,j|K}\Delta) - \frac{n_{k,j}}{\lambda_{k,j|K}^2}\left(\frac{\partial \lambda_{k,j|K}}{\partial x_k}\right)^2 \right]\mathbb{E}\big[x_k - x_{k|K}\big]^2$$
$$\approx \log(\lambda_{k,j|K}\Delta)n_{k,j} - \lambda_{k,j|K}\Delta + 0$$

$$+\frac{1}{2}\left[\frac{1}{\lambda_{k,j|K}}\frac{\partial^2\lambda_{k,j|K}}{\partial x_k^2}(n_{k,j}-\lambda_{k,j|K}\Delta)-\frac{n_{k,j}}{\lambda_{k,j|K}^2}\left(\frac{\partial\lambda_{k,j|K}}{\partial x_k}\right)^2\right]\sigma_{k|K}^2. \tag{39}$$

Therefore,

$$Q_6 \approx \sum_{k=1}^{K}\sum_{j=1}^{J}\log(\lambda_{k,j|K}\Delta)n_{k,j}-\lambda_{k,j|K}\Delta+\frac{1}{2}\left[\frac{1}{\lambda_{k,j|K}}\frac{\partial^2\lambda_{k,j|K}}{\partial x_k^2}(n_{k,j}-\lambda_{k,j|K}\Delta)-\frac{n_{k,j}}{\lambda_{k,j|K}^2}\left(\frac{\partial\lambda_{k,j|K}}{\partial x_k}\right)^2\right]\sigma_{k|K}^2. \tag{40}$$

2 Experimental data – model parameter estimates

The experimental model parameters estimated for each participant are shown in Table 1. Recall that we estimate x_k at the E-step and calculate the model parameters at the M-step. Recall also that due to computational complexity we split the estimation of the model parameters related to heart rate (i.e., the θ_i's and the η coefficient) into two parts and calculate them separately. We calculate the θ_i's offline based on maximum likelihood estimation (MLE) and select η based on which value maximized a log-likelihood term. As pointed out in the "Discussion" section of the main text, this separated-out calculation is a limitation of our model (e.g. it can result in numerical issues). The separate estimation of the heart rate parameters may be the reason why η values are small in the final estimates and why larger η values cause convergence issues in the Newton-Raphson solution for the state update $x_{k|k}$ since the MLE estimation of the θ_i's may account for much of the heart rate variability. The value of β_1 also turned out to be negative for two participants (the M-step updates turned out to be negative even after the first iteration). Our algorithm provides two options for calculating β_0 and β_1 and it is possible to select the alternate option which sets $\beta_0 = 1$ and calculates β_1 empirically as well if this is to be avoided beforehand. As noted in the main text, a model with a less complex form of the conditional intensity function may enable all the parameters to be recovered at once at the M-step. Lower computational load is also likely to have the benefit of easier deployment onto a wearable platform.

Table 1: **Estimated model parameters on experimental data**

Participant	α	ρ	δ_0	δ_1	σ_w^2	γ_0	γ_1	σ_v^2	β_0	β_1	σ_ε^2	η
1	0.1339	0.9964	6.0453	0.8501	0.4475	-2.1516	0.8182	0.4881	-4.5533	0.1642	0.0147	-10^{-4}
2	0.1752	0.9984	10.4601	0.8507	0.4731	-6.3457	0.8019	0.5319	-4.9209	0.1145	0.0149	-10^{-6}
3	0.2050	0.9944	15.4847	0.9157	0.5967	-2.9270	0.8805	0.6270	-3.9055	0.2620	0.0196	-10^{-6}
4	0.2122	0.9889	14.8302	0.9337	0.5191	-3.3621	0.9018	0.5515	-3.1620	0.0282	0.0195	-10^{-6}
5	0.1779	0.9963	13.4671	0.8726	0.4388	-3.6769	0.7916	0.5381	-3.7924	-0.1577	0.0150	-10^{-6}
6	0.1532	0.9888	14.6993	0.8869	0.4876	-0.5512	0.8542	0.5247	-3.8582	0.1047	0.0170	-10^{-5}
7	0.0934	0.9986	4.0806	0.8944	0.2311	-2.6035	0.8653	0.2804	-4.1830	0.5256	0.0109	-10^{-3}
8	0.1231	0.9933	13.5384	0.8755	0.4095	-0.9875	0.8193	0.4829	-3.6242	0.3115	0.0148	-10^{-4}
9	0.1587	0.9966	6.0052	0.9050	0.3256	-4.0313	0.8408	0.4178	-3.6640	-0.1002	0.0141	-10^{-6}
10	0.1365	0.9911	5.6286	0.8804	0.3722	-2.1238	0.8515	0.4127	-4.3647	0.1101	0.0142	-10^{-4}
11	0.1533	0.9947	17.6648	0.9119	0.4269	-0.7794	0.8601	0.4901	-3.4974	0.1716	0.0171	-10^{-3}
12	0.1536	0.9931	11.5030	0.9069	0.3077	-1.6278	0.8558	0.3835	-4.0583	0.2806	0.0140	-10^{-6}

Participant	θ_i's
1	0.0744, 1.1497, -0.1054, -0.1686, 1289.5
2	0.0733, 1.0793, -0.2674, -0.0028, 0.4085, -0.4041, -0.0461, 0.1289, 0.0149, 1404.5
3	0.2484, 0.7572, 231.5820
4	0.2079, 1.3273, -0.4968, -0.0615, -0.0765, -0.0068, 0.0194, 341.5199
5	0.1319, 1.3161, -0.4226, -0.1202, 0.0481, -0.0603, 0.0376, 561.4294
6	0.0677, 0.9083, 625.1079
7	0.1478, 1.0039, -0.2359, 0.0760, 585.6449
8	0.1289, 1.1844, -0.3928, 699.4618
9	0.0229, 0.7706, -0.0228, -0.1910, 0.2809, 0.1220, 1253.4
10	0.1575, 1.1033, -0.3104, 714.4876
11	0.0990, 1.0544, -0.4583, 0.0098, 0.1616, 0.0254, -0.0710, 0.0540, 0.1015, 107.0561
12	0.0818, 0.7980, -0.2500, 0.0853, 0.2650, 693.1574

References

[1] Coleman TP, Yanike M, Suzuki WA, Brown EN. A mixed-filter algorithm for dynamically tracking learning from multiple behavioral and neurophysiological measures. In: The dynamic brain: An exploration of neuronal variability and its functional significance. Oxford Univ. Press; 2011. p. 1–16.

[2] Smith AC, Frank LM, Wirth S, Yanike M, Hu D, Kubota Y, et al. Dynamic analysis of learning in behavioral experiments. Journal of Neuroscience. 2004;24(2):447–461.

[3] Prerau MJ, Smith AC, Eden UT, Kubota Y, Yanike M, Suzuki W, et al. Characterizing learning by simultaneous analysis of continuous and binary measures of performance. Journal of Neurophysiology. 2009;102(5):3060–3072.
